# Discrete Time Series Forecasting of Hive Weight, In-Hive Temperature, and Hive Entrance Traffic in Non-Invasive Monitoring of Managed Honey Bee Colonies: Part I

**DOI:** 10.3390/s24196433

**Published:** 2024-10-04

**Authors:** Vladimir A. Kulyukin, Daniel Coster, Aleksey V. Kulyukin, William Meikle, Milagra Weiss

**Affiliations:** 1 Department of Computer Science, Utah State University, Logan, UT 84322, USA; 2 Department of Mathematics and Statistics, Utah State University, Logan, UT 84322, USA; 3 Department of Data Analytics and Information Systems, Huntsman School of Business, Utah State University, Logan, UT 84322, USA; 4 Carl Hayden Bee Research Center, United States Department of Agriculture, Agricultural Research Service, Tucson, AZ 85719, USA

**Keywords:** discrete time series forecasting, predictive hive monitoring, hive monitoring sensors, precision apiculture, FAIR datasets, artificial neural networks, convolutional neural networks, long short-term memory, autoregressive integrated moving average, ARIMA

## Abstract

From June to October, 2022, we recorded the weight, the internal temperature, and the hive entrance video traffic of ten managed honey bee (*Apis mellifera*) colonies at a research apiary of the Carl Hayden Bee Research Center in Tucson, AZ, USA. The weight and temperature were recorded every five minutes around the clock. The 30 s videos were recorded every five minutes daily from 7:00 to 20:55. We curated the collected data into a dataset of 758,703 records (280,760–weight; 322,570–temperature; 155,373–video). A principal objective of Part I of our investigation was to use the curated dataset to investigate the discrete univariate time series forecasting of hive weight, in-hive temperature, and hive entrance traffic with shallow artificial, convolutional, and long short-term memory networks and to compare their predictive performance with traditional autoregressive integrated moving average models. We trained and tested all models with a 70/30 train/test split. We varied the intake and the predicted horizon of each model from 6 to 24 hourly means. Each artificial, convolutional, and long short-term memory network was trained for 500 epochs. We evaluated 24,840 trained models on the test data with the mean squared error. The autoregressive integrated moving average models performed on par with their machine learning counterparts, and all model types were able to predict falling, rising, and unchanging trends over all predicted horizons. We made the curated dataset public for replication.

## 1. Introduction

Many studies have documented significant declines of domesticated and wild pollinators worldwide (cf., e.g., Potts et al., 2010 [1]; Van Klink et al., 2020 [2]; Woodard et al., 2021 [3]). Since in the U.S. the honey bee (*Apis mellifera*) remains an important pollinator, the U.S. government developed a strategy to promote the health of the honey bee in the public document titled *The National Strategy to Promote the Health of Honey Bees and Other Pollinators* [4]. Tracking the health and status of managed colonies requires continuous monitoring. Human monitoring is difficult because beekeepers have limited time, patience, and resources. Sensor-based monitoring can alleviate the bottleneck (cf., e.g., Buchmann and Thoenes, 1990 [5]; Thoenes and Buchmann, 1992 [6]; Marceau et al., 1991 [7]; Odemer, 2021 [8]; Tashakkori et al., 2021 [9]). However, for such monitoring to become useful, two key challenges must be addressed: (1) lack of **F**indable, **A**ccessible, **I**nteroperable, and **R**eusable (FAIR) (Wilkinson, Dumontier, Aalbersberg et al., 2016 [10]) multisensor datasets for precision apiculture and (2) insufficient predictive modeling.

In precision apiculture, FAIR multisensor, longitudinal datasets across multiple geographical locations, field experiments, and bee races do not exist (cf., e.g., Zaman and Dorin (2023) [11]), which constitutes a major barrier to progress because such datasets could catalyze research and inform practice (cf., e.g., Kulyukin, 2021 [12]). Predictive modeling relies on machine learning (ML), a branch of computer science that focuses on solving problems for which the development of algorithms by human programmers may not be cost-effective (Mitchell, 1997 [13]) or on statistical models such as autoregressive integrated moving average (ARIMA) and variants thereof (cf., e.g., Bowerman and O’Connell, 1993 [14]). Predictive modeling for precision apiculture is in its infancy (cf., e.g., Zaman and Dorin (2023) [11]) in that computational models that reliably forecast the status of managed colonies from sensors deployed in and around the hive are few and far between, especially models that align sensor measurements with hive inspections executed according to rigorous hive management protocols (Braga et al., 2020 [15]). The principal causes of this state of affairs are varied and include, but may not be limited to, hardware sensor failures, natural calamities that destroy sensors and colonies, and social and economic difficulties associated with finding apiaries with sufficient numbers of managed colonies whose owners are willing to install sensors for long periods of time. These causes preclude apiary science researchers from creating sufficiently large FAIR datasets on which predictive models for precision apiculture can be compared with each other.

Our contributions to state-of-the-art precision apiculture and sensor-based monitoring of managed hives reported in this article are as follows. First, we curated a dataset of 758,703 records (280,760 weight; 322,570 temperature; 155,373 entrance traffic video). Second, we organized the dataset according to the FAIR principles and made it publicly available as a precision apiculture benchmark in our supplementary materials. We did not find FAIR datasets of comparable size and coverage in the precision apiculture and continuous hive monitoring literature we had reviewed for our investigation. Third, we constructed and evaluated 24,840 shallow artificial neural network (ANN), convolutional neural network (CNN), long short-term memory (LSTM), and traditional autoregressive integrated moving average (ARIMA) models and included our source code in the FAIR dataset for replication. Our principal objective was to use the curated dataset to investigate discrete univariate time series (DUTS) forecasting of hive weight, in-hive temperature, and hive entrance traffic with the three machine learning (ML) models (i.e., ANN, CNN, and LSTM) and to compare their predictive performance with ARIMA. Our selection of these ML models was motivated by the fact that they remain the state-of-the-art architectures for time series analysis in many areas of data science, such as text and audio classification (cf., e.g., Fawaz et al., 2019 [16]) and prediction of physiological signals in clinical trials (cf., e.g., Pham, 2021 [17]. To our knowledge, this is the first attempt to construct DUTS forecasters of hive weight, in-hive temperature, and hive entrance traffic for precision apiculture with these ML models and to compare them with ARIMA. Fourth, we experimentally discovered that the mean hourly hive weight, in-hive temperature, and hive entrance traffic of all 10 colonies for which we collected the data could be predicted with a reasonable degree of accuracy on the time spans of 12, 24, and 48 h and that the ARIMA forecasters performed on par with their ANN, CNN, and LSTM counterparts, which has theoretical and practical implications for multisensor hive monitoring.

The remainder of our article is organized as follows. In Section 2, we review related research. In Section 3, we describe our metadata, data, and methods to construct and evaluate the forecasters. In Section 4, we present the results of our evaluation. In Section 5, we discuss our results in the broader context of multisensor precision apiculture systems and predictive hive monitoring and outline some theoretical and practical implications of our findings. In Section 6, we offer our conclusions and outline the planned scope of Part II of this investigation.

## 2. Related Work

Many apiary scientists have used scales to characterize colony events through weight data. Buchmann and Thoenes (1990) [5] and Thoenes and Buchmann (1992) [6] showed that the colony weight is related to its foraging, swarming, and hive abandonment. Marceau et al. (1991) [7] demonstrated a polynomial regression fit between hive weight and colony growth, consumption, and productivity. Meikle et al. (2006) [18] and Zacepins et al. (2016) [19] partially corroborated Buchmann and Thoenes’ findings on weight and swarming. Meikle et al. (2008) [20] showed that within-day variation of hive weight could be used as a measure of colony activity. Meikle et al. (2016) [21] demonstrated that hive weight and in-hive temperature could be used to monitor colony phenology and investigated a relationship between colony weight and exposure to pesticides. Stalidzans et al. (2017) [22] found a relationship between colony weight and overwintering.

In-hive temperature sensors also provide data relevant to the status of a managed colony. These small sensors are placed inside a hive, e.g., on the wall of a hive super or in the middle of an individual frame. Szabo et al. (1989) [23] demonstrated that when in-hive temperature sensors were placed inside or close to the mass of bees at the core of the colony, which the researchers called the *cluster*, the sensors were affected more by the cluster and less by exterior conditions than the sensors placed further from the cluster. Southwick and Moritz (1987) [24] experimentally showed the possibility that daily cycles of in-hive temperature and metabolic activity are driven by ambient conditions. Separated by almost a century, the field investigations by Gates [25] (1914) and Meikle et al. (2016) [26] reported some evidence of the thermoregulation of colonies. Meikle et al. (2016) [26] showed that in-hive temperature is affected by colony size and the location of the in-hive temperature sensor. Worswick (1987) [27] argued that the intensity of the colony’s thermoregulation was a function of subspecies. Jones et al. (2004) [28] provided evidence that thermoregulation may be related to the within-colony genetic diversity. The hypothesis advocated by Stalidzans and Berzonis (2013) [29] is that thermoregulation depends on the colony’s phenological status. Meikle et al. (2018) [30] observed the codependencies between thermoregulation and pesticide exposure.

Bee traffic at the hive’s entrance, which we will hereafter call *bee entrance traffic* or simply *entrance traffic*, has been investigated with cameras for almost a century. Patterson (1935) [31] designed an image-based bee counter in 1935 by means of a wide-angle lens and 35 mm film. Single bee passes were manually counted as crossings of a line in the image. For the next 60 years, image and video sensors were not part of the insect motion literature until the appearance of digital cameras in the late 1990’s, when Dickinson et al. (1999) [32] used digital images to investigate the aerodynamics of insect flight. Chen et al. (2012) [33] recorded videos of bees illuminated with infrared light at the hive entrance. Bees were individually marked with special characters identifiable with Hough transform to quantify some aspects of entrance traffic. Dussaubat et al. (2013) [34] designed similar techniques to investigate the effects of *Nosema ceranae* infection on the flight behavior of bees.

A recent trend in image- and video-based entrance traffic quantification is the enhancement of methods of standard ML with deep learning (DL) and computer vision. DL is a branch of artificial intelligence (AI) that focuses on the design and application of convolutional neural networks (CNNs) to problems varying from classification to regression (cf., e.g., Thompson et al. (2021) [35]). Chiron et al. (2013) [36] proposed a 3D stereo vision algorithm to detect and track honey bees at the hive’s entrance. Babic et al. (2016) [37] and Yang et al. (2017) [38] used ML and DL methods to differentiate between incoming pollen- and nonpollen-bearing foragers. Tashakkori et al. (2021) [9] used computer vision techniques to estimate the number of drones in a managed hive. Kulyukin et al. (2022) [39] experimentally demonstrated the possibility of a relationship between the hive weight and the video-based measurements of entrance traffic. Kulyukin and Kulyukin (2023) [40] combined motion detection with DL-based bee object inference to quantify omnidirectional entrance traffic in videos. Hamza et al. (2023) [41] proposed to use a camera above the hive’s entrance to record entrance traffic in a BeeLive platform for their Beemon hive monitoring system. What unifies these investigations is the ultimate objective of designing algorithms to quantify various characteristics of entrance traffic from videos.

Another incipient and growing trend is the predictive modeling of various characteristics of managed colonies. Braga et al. (2020) [15] designed several computer models of hive distress detection and prediction based on a comprehensive colony checklist. In a 3-year-long investigation of multiple colonies at multiple locations, the researchers used the internal hive temperature, hive weight, ambient temperature, dew point, wind direction, wind speed, rainfall, and daylight in combination with weekly apiary inspection results. K-nearest neighbors (KNNs) models, random forests, and ANNs were trained to predict hive health from the internal temperature, weight, and ambient weather. On the collected dataset, a random forest turned out to be the best predictor with an accuracy of 98%. Zaman and Dorin (2023) [11] proposed a theoretical framework for predictive hive monitoring that takes into account the interests and objectives of different stakeholders who stand to benefit from it.

In conducting the background research on predictive modeling, we found evidence of predictive modeling on the websites of four commercial multisensor platforms: *Arnia* (model: *Arnia Perfetta*™; url: www.arniaperfetta.it (accessed on 20 September 2024))—8 sensors: audio, temperature, humidity, weight, light sensor, accelerometer, bee counter, video; *ApisProtect* (model: *ApisProtect 2023*; url: linkedin.com/company/apisprotect (accessed on 20 September 2024))—4 sensors: temperature, humidity, audio, accelerometer; *IoBee* (model: *IoBee SOA*; url: io-bee.eu (accessed on 20 September 2024))—4 sensors: temperature, humidity, weight, bee counter; *Pollenity* (model: *Pollenity Merchant*; url: www.pollenity.com (accessed on 20 September 2024))—4 sensors: temperature, humidity, weight, acoustic. We could not analyze the predictive power of these platforms because the software tools appear to be proprietary. Nor did we find evidence of FAIR datasets on these commercial sites.

Another four multisensor commercial platforms that we came across during our background research are: *BuzzBox* (model: *BuzzBox Hive Health Monitor*; url: www.beebuilt.com (accessed on 20 September 2024))—3 sensors: temperature, humidity, audio; *BroodMinder* (model: *BroodMinder Apiary Starter Pack*; url: broodminder.com (accessed on 20 September 2024))—3 sensors: temperature, humidity, weight; *Hive Mind* (model: *HiveMind Hive Strength Monitor*; url: hivemind.nz (accessed on 20 September 2024))—5 sensors: temperature, humidity, weight, bee counter, rain gauge; *Hyper Hyve* (model: *HyperHive*™; url: hyperhyve.com (accessed on 20 September 2024))—3 sensors: temperature, humidity, weight). However, these platforms appear to focus on remote visualization of sensor data and leave the interpretation to the human user. It should be noted that the sites of commercial platforms are volatile insomuch as the information on the available product models is constantly added, updated, and deleted.

## 3. Materials and Methods

### 3.1. Metadata

**Figure 1:**
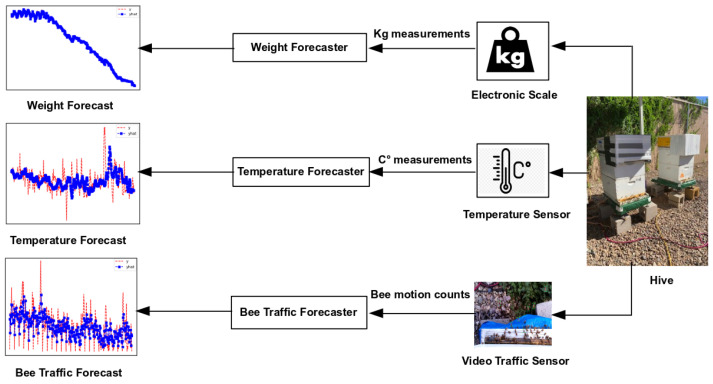
**Data pipeline.** Right to left: a hive mounted on an electronic scale (weight sensor) with an in-hive temperature sensor (cf. Figure A3) and an external on-hive video sensor. The weight sensor generates a time series of kg weight measurements (real numbers); the temperature sensor—a time series of °C temperature measurements (real numbers); the video traffic sensor—a time series of bee motion counts (natural numbers). The three series are time-aligned.

**Figure A1:**
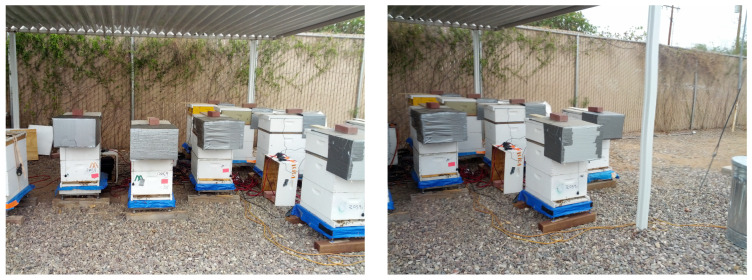
**Research Apiary.** Two views of the USDA-ARS research apiary in Tucson, AZ, USA (GPS coordinates: 32°13′18.274″ N, 110°55′35.324″ W), where the hive weight, in-hive temperature, and video entrance traffic data were collected from 10 hives from June to October, 2022. The large light gray boxes on the monitored hives protected cameras against the elements.

We acquired the data for this investigation from 10 colonies in Langstroth hives at a research apiary of the Carl Hayden Bee Research Center of the U.S. Department of Agriculture Agricultural Research Service (USDA-ARS) in Tucson, Arizona (AZ), USA (GPS coordinates: 32°13′18.274″ N, 110°55′35.324″ W) from June to October, 2022. The archived weather conditions for this time period are available from the Arizona Meterological Network of the University of Arizona College of Agriculture and Life Sciences [42]. Each hive was mounted on an electronic scale and consisted of a bottom board with a landing pad, two deep Langstroth boxes with 10 frames in each with an in-hive temperature sensor installed in the middle frame of the second (higher) box, an inner hive cover, a box with an on-hive video traffic sensor, and a hive cover with a cardboard box reinforced with all-weather duct tape to protect the camera against the elements (cf. Figure 1 and Figure A1).

**Figure A3:**
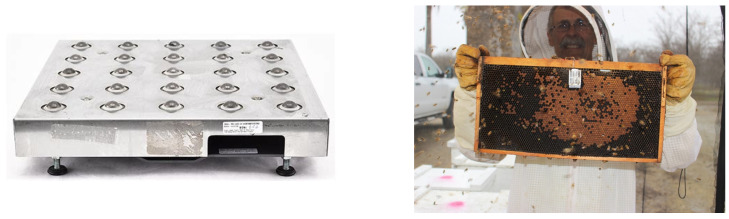
**Weight and temperature Sensors. LEFT**: Tekfa electronic scale B-2418. **RIGHT**: an internal wireless temperature sensor (Hobo MX2201) placed at the top bar of the middle frame in the second (higher) box of each monitored hive with one temperature sensor per hive. Since the temperature sensor is placed inside the hive, it is not affected by sunlight.

From 9 June to 14 June 2022, each hive was placed on a stainless steel electronic scale (Tekfa model B-2418, precision: ±20 g; operating temperature: −30 °C to 70 °C) linked to a 16-bit datalogger (Hobo UX120-006M External Channel data logger, Onset Computer Corporation, Bourne, MA, USA). On 21 June 2022, an in-hive temperature sensor (Hobo MX2201 sensor, Onset Computer Corporation, Bourne, MA; accuracy ± 0.5 °C) was placed at the top bar of the middle frame in the second box of each hive (cf. Figure A3). Ten BeePi on-hive video loggers (vloggers) (cf., e.g., Kulyukin et al., 2022 [39]) were installed on 23 June 2022. Each vlogger was equipped with a Raspberry Pi 3 model B v1.2 computer coupled to a Raspberry Pi v2 camera (8 megapixel, 1080 × 1920 pix resolution, 25 frames per second (fps)) that looked down on the landing pad of the hive from the top of the second box. The installation software and hardware quality evaluation were conducted for each vlogger on 24 June 2022. The evaluation involved a visual verification of the correctness of the hardware setup, a wireless login into the Raspberry Pi computer of each vlogger via an ad hoc Wi-Fi network, a secure retrieval of several captured videos from a USB hardware disk connected to each vlogger, and a viewing of each retrieved MP4 RGB video in a video player. The scales and the vloggers were powered from the grid. Each in-hive temperature sensor had its own battery the size of a small coin that could power it for ≈12 months.

Five hives had Russian queens and five hives had Wooten queens (cf. Table 1). Hive evaluations were conducted on 21 June, 11 August, and 23 September. Each evaluation included a visual queen status check (presence/absence) and removal of queen supersedure cells. The weight of the hive woodenware was not affected by rainfall because the June–October weather in Tucson, AZ, is hot (≥35 °C) and dry with almost no rainfall [42]. Rapid hive inspections to check queen status and hive strength were conducted on 22 July and 16 September. A new Russian queen was installed in hive 2141 on 20 June 2022, and a new Wooten queen in hive 2140 on 23 June 2022. On 22 July 2022, a supersedure queen cell was removed from 2059. On 26 July 2022, a new Russian queen was again installed in hive 2141, and a new Russian queen was installed in hive 2059. On 15 August 2022, a laying worker was detected in hive 2141. On 19 September 2022, the queens were removed from hives 2158 and 2120 for other scheduled experiments unrelated to this investigation. The final hive evaluations were conducted on 23 September 2022. On 28 September 2022, the vloggers were removed from 2137 and 2146 because the hives were scheduled to move to a different laboratory. All BeePi units were disassembled on 11 October 2022.

### 3.2. Data

The total numbers of logged records for each sensor are detailed in Table 1. We time- aligned the weight, temperature, and video data by their time stamps and smoothed the weight and temperature data by computing hourly means for each hive. We computed the omnidirectional bee traffic counts for each video. The traffic counts are natural numbers, i.e., non-negative integers, of flying bees detected in each frame of a video. The omnidirectional bee traffic counts were computed with our OmniBeeM (Omnidirectional Bee Motion) algorithm (cf., e.g., Kulyukin and Mukherjee, 2019 [43]). For each video, OmniBeeM returns three sets of objects: (1) motion regions (motion rectangles); (2) inferred bee objects aligned with motion regions (motion-aligned bee rectangles); and (3) motion-unaligned inferred bee objects (unaligned bee rectangles). The cardinality of the set of the motion-aligned bee rectangles (a non-negative integer) is returned as the omnidirectional traffic estimate for the video. The algorithm is agnostic to motion detection methods and bee object inference methods insomuch as it can work with DL and non-DL object inference models (cf. Kulyukin et al., 2021 [44]). In this investigation, we computed the omnidirectional traffic counts for each video with OmniBeeM working with our YOLOv3, YOLOv4-Tiny, and YOLOv7-Tiny models we trained in our previous research to recognize flying bee objects in videos (cf., e.g., Kulyukin and Kulyukin, 2023 [40] for details). We then computed the hourly traffic means for each hive. Finally, we aligned all hourly means with natural numbers to obtain the same time axis for the time series analysis and saved these records in the CSV files provided in the supplementary materials. We used these time-aligned hourly means as the ground truth measurements for the three DUTS: (1) weight series denoted as $\left\{W_{t}\right\}$; (2) temperature series denoted as $\left\{C_{t}\right\}$, because the temperature was recorded in degrees Celsius; and (3) bee entrance traffic series denoted as $\left\{B_{t}\right\}$. Table 2 gives a sample record of hourly means. Table 3 gives total numbers of hourly means for each hive used in the DUTS analysis.

### 3.3. Discrete Univariate Time Series Forecasting

A DUTS is a set of observations $\left\{o_{t}|t\in T_{X_{t}}\right\}$, where each $o_{t}$ is recorded at time *t* and $T_{X_{t}}$ is a set of discrete time values of a random variable $X_{t}$. The term *univariate* means that $o_{t}$ is a value of exactly one random variable. In our investigation, the three random variables were the hive weight $W_{t}$, the in-hive temperature $C_{t}$, and the bee entrance traffic $B_{t}$ such that

(1) $$\begin{array}{ccccccc} W_{t} & \in & \{w_{t_{1}},\ldots w_{t_{k}}\}, & 0<k\in N, & w_{t_{j}}\in R,\; & t_{j}\in T_{W}\subset N, & 1\leq j\leq k;\\ C_{t} & \in & \{c_{t_{1}},\ldots c_{t_{m}}\}, & 0<m\in N, & c_{t_{j}}\in R,\; & t_{j}\in T_{C}\subset N, & 1\leq j\leq m;\\ B_{t} & \in & \{b_{t_{1}},\ldots b_{t_{n}}\}, & 0<n\in N, & b_{t_{j}}\in R,\; & t_{j}\in T_{B}\subset N, & 1\leq j\leq n, \end{array}$$

where ⊂ denotes the proper subset relation between two sets, R denotes the set of real numbers, N denotes the set of natural numbers, and t∈N denotes a unique natural number corresponding to a time stamp. For each sensor, we defined a 1–1 map whose domain was a finite set of the sensor’s digital clock time stamps (year, month, day, hour, minutes, seconds) and whose range was a finite subset of N. $T_{W}$, $T_{C}$, and $T_{B}$ were constructed as the ascending ranges of the appropriate maps. $\left\{W_{t}\right\}$ and $\left\{C_{t}\right\}$ were completely time-aligned for each hive, i.e., m=k and $T_{W}=T_{C}$. $\left\{B_{t}\right\}$ was partially time-aligned with $\left\{W_{t}\right\}$ and $\left\{C_{t}\right\}$ for each hive insomuch as no videos were captured during the night hours, i.e., n<m, n<k, $T_{B}\subset T_{W}$, $T_{B}\subset T_{C}$.

We constructed three types of DUTS forecasters: weight forecasters ${\hat{F}}_{M,W_{t},n,k}$, temperature forecasters ${\hat{F}}_{M,C_{t},IN,OUT}$, and traffic forecasters ${\hat{F}}_{M,B_{t},n,k}$. The first subscript *M* denotes a model through which the forecaster was realized, i.e., *A* for ANN, *C* for CNN, *L* for LSTM, *R* for ARIMA. The second subscript, i.e., $W_{t}$, $C_{t}$, $B_{t}$, refers to the random variable in (1) predicted by the forecaster. The third and forth subscripts (i.e., IN and OUT or, equivalently, *n* and *k* in the notation of (2)) refer to the input and output of the forecater. Specifically, a forecaster with 0<IN=n∈N and 0<OUT=k∈N in (2) maps an *n*-tuple of observed real values (e.g., *n* hourly bee traffic means $b_{i_{1}},\ldots ,b_{i_{n}}$) to a *k*-tuple of predicted values (e.g., *k* predicted hourly bee traffic means ${\hat{b}}_{i_{n+1}},\ldots ,{\hat{b}}_{i_{n+k}}$). We will hereafter call the value of IN (or, equivalently, of *n*) the *intake* and the value of OUT (or, equivalently, of *k*) the *predicted horizon* or simply *horizon*. In short, the subscripts *n* and *k*, respectively, denote the IN and OUT values for each forecaster or the forecaster’s intake and the horizon. Table 4 gives the investigated intake and horizon values. We use the term *model* to refer to a specific implementation of a forecaster. We use the term *time span* or simply *span* to refer to the sum of the forecaster’s IN and OUT values.

(2) $$\begin{array}{cccc} {\hat{F}}_{M,W_{t},n,k}:R^{n}\mapsto R^{k}; & {\hat{F}}_{W_{t}}\left(w_{i_{1}},\ldots ,w_{i_{n}}\right) & = & {\hat{w}}_{i_{n+1}},\ldots ,{\hat{w}}_{i_{n+k}};\\ {\hat{F}}_{M,C_{t},n,k}:R^{n}\mapsto R^{k}; & {\hat{F}}_{C_{t}}\left(c_{i_{1}},\ldots ,c_{i_{n}}\right) & = & {\hat{c}}_{i_{n+1}},\ldots ,{\hat{c}}_{i_{n+k}};\\ {\hat{F}}_{M,B_{t},n,k}:R^{n}\mapsto R^{k}; & {\hat{F}}_{B_{t}}\left(b_{i_{1}},\ldots ,b_{i_{n}}\right) & = & {\hat{b}}_{i_{n+1}},\ldots ,{\hat{b}}_{i_{n+k}}. \end{array}$$

For instance, let $M\in \{M_{A},M_{C},M_{L},M_{R}\}$, where $M_{A}$, $M_{C}$, $M_{L}$, and $M_{R}$ respectively denote ANN, CNN, LSTM, and ARIMA. Then, with the notation of (1) and (2), we can define three forecasting models: ${\hat{F}}_{M_{A},W_{t},6,2}$, ${\hat{F}}_{M_{L},C_{t},12,9}$, and ${\hat{F}}_{M_{R},B_{t},24,18}$. ${\hat{F}}_{M_{A},W_{t},6,2}$ uses $M_{A}$ to predict k=2 mean hourly weight observations from the previously observed n=6 mean hourly weight measurements. In the notation of Table 4, the intake of this model is IN = 6 h, the horizon is OUT =2 h, and the span is IN + OUT = 6 + 2 = 8 h. Analogously, ${\hat{F}}_{M_{L},C_{t},12,9}$ uses $M_{L}$ to predict k=9 mean hourly temperature values from the previously observed n=12 mean hourly temperature values. The intake of this forecaster is IN = 12 h, the horizon is OUT = 9 h, and the span is IN + OUT = 12 + 9 = 21 h. Finally, ${\hat{F}}_{M_{R},B_{t},24,18}$ uses $M_{R}$ to predict k=18 mean hourly traffic counts from the previously observed n=24 mean hourly bee traffic counts. The intake of this forecaster is IN = 24 h, the horizon is OUT = 18 h, and the span is IN + OUT = 24 + 18 = 42 h. For brevity, we will sometimes refer to forecasters and models by their IN and OUT values. For example, IN = 6, OUT = 2, or simply 6, 2 ARIMA weight forecaster, or IN = 24, OUT = 12, or 24, 12 LSTM traffic model. We will also use phrases such as IN = 6 forecasters to refer to forecasters with an intake of 6 h. We will evaluate the performance of ${\hat{F}}_{M,V,IN,OUT}$ on a sequence of test observations $S_{test}=o_{t_{1}},\ldots ,o_{t_{l}}$, l>1, with a mean squared error (MSE) function

(3) $$\begin{array}{ccc} MSE\left({\hat{F}}_{M,V,OUT,IN},S_{test}\right) & = & \frac{1}{l-sp+1}\sum_{e\in E}^{l}{\left(o_{t_{e}}-{\hat{o}}_{t_{e}}\right)}^{2}, \end{array}$$

where sp is the forecaster’s span, E={s+sp−1|1≤s≤l−sp+1}, and ${\hat{o}}_{t_{e}}$ is the forecaster’s prediction at time $t_{e}$.

### 3.4. Construction and Evaluation of Forecasting Models

**Figure A6:**
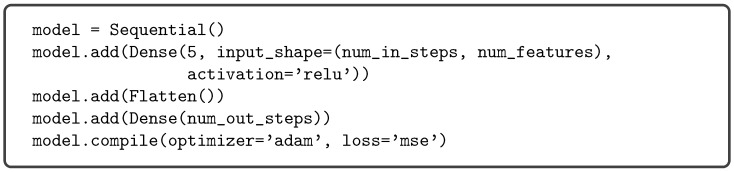
**ANN forecaster.** The artificial neural network (ANN) $M_{A}$ in each forecaster ${\hat{F}}_{M_{A},V,IN,OUT}$. Since we performed univariate time series forecasting, num_features = 1; num_in_steps and num_out_steps assumed the values of intake and horizon, respectively, in Table 4. The input layer feeds into a fully connected, i.e., dense, layer of 5 ReLU-activated nodes. The output of the dense layer is flattened, i.e., turned into a 1D vector, and fed into a fully connected layer with the number of nodes specified by num_out_steps. $M_{A}$ is compiled with the Adam optimizer function and the mean squared error (MSE) loss function.

**Figure A7:**
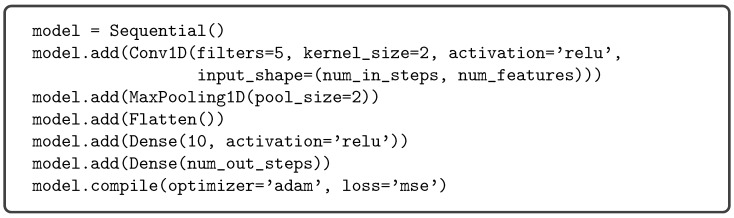
**CNN forecaster.** The convolutional neural network (CNN) $M_{C}$ in each forecaster ${\hat{F}}_{M_{C},V,IN,OUT}$. In $M_{C}$, num_features = 1; num_in_steps and num_out_steps are the values in Table 4. The input layer of $M_{C}$ feeds into a convolutional layer with 5 filters and kernel size of 2; the nodes of the convolutional layer are activated with ReLU. The convolutional layer feeds into a max pooling layer with a pool size of 2. The output of the max pooling layer is flattened and fed into a fully connected, i.e., dense, layer of 10 ReLU-activated nodes. The fully connected layer feeds into another fully connected layer of num_out_steps nodes. Each $M_{c}$ is compiled with the Adam optimizer function and the mean squared error (MSE) loss function.

**Figure A8:**
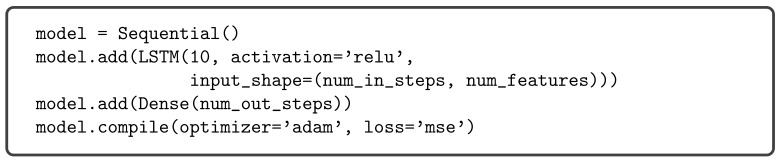
**LSTM forecaster.** The long short-term memory (LSTM) model $M_{L}$ in ${\hat{F}}_{M_{L},V,IN,OUT}$. In $M_{L}$, num_features = 1, and num_in_steps and num_out_steps assume the values in Table 4. The input layer of $M_{L}$ feeds into an LSTM layer with 10 ReLU-activated nodes. The output of the LSTM layer feeds into a fully connected layer with num_out_steps nodes. Each $M_{L}$ is compiled with the Adam optimizer function and the mean squared error (MSE) loss function.

We implemented each ML forecaster in Python 3.10 with the Keras library on Ubuntu 22.04 LTS (cf. the source code in Figure A6, Figure A7 and Figure A8). In traffic forecasters, we used only the bee motion counts obtained with OmniBeeM using YOLOv3 as a bee object inference model because we plan to perform a comparative analysis of the traffic forecasters of the OmniBeeM counts with the YOLOv3, YOLOv4-Tiny, and YOLOv7-Tiny bee inference models in Part II of our investigation (cf. Section 6).

For each $\left\{X_{t}\right\}=\left\{x_{t}|t=1,\ldots ,K\right\}$, where X∈{W,T,B}, x∈{w,c,b}, and K∈N (cf. Equation (1)), we constructed an ARIMA model in SAS 9.4 (SAS Institute Inc., Cary, NC, USA) using IN =K with the following two steps recommended by Bowerman and O’Connell (1993) [14]. We achieved the first order stationarity and approximated stationarity by computing the first differenced series

(4) $$\begin{array}{cccc} z_{t} & = & x_{t}-x_{t-1}, & 2\leq t\leq K, \end{array}$$

and then constructed an autoregressive model of order k−1, denoted AR(k−1), to the first differenced series $\left\{z_{t}\right\}$ as

(5) $$\begin{array}{c} z_{t}-\phi_{1}z_{t-1}-\phi_{2}z_{t-2}\cdots -\phi_{k-1}z_{t-(k-1)}=\delta +a_{t}, \end{array}$$

where $\left\{\phi_{1},\phi_{2},\ldots ,\phi_{k-1}\right\}$ are the k−1 autoregressive parameters, δ is a fixed constant, usually close to 0, and $\left\{a_{t}\right\}$ are assumed to be independent random shocks with a mean of 0.

For each hive, we did a 70/30 train/test split on the number of the observed mean values of $W_{t}$, $C_{t}$, and $B_{t}$ (cf. Table 3) and trained each ML model (i.e., $M_{A}$, $M_{C}$, $M_{L}$ in Figure A6, Figure A7 and Figure A8) for each possible IN, OUT pair in Table 4 and each possible value of $V\in \{W_{t},C_{t},B_{t}\}$ 5 times for 500 epochs. Thus, we trained each of the possible 24,840 models 5 times for 500 epochs, where the number 24,840 is computed as follows: 828 is computed from Table 4 as $3\times (6^{2}+12^{2}+24\times 4)$ and then multiplied by 3, i.e., the number of the possible values of *V*, and then by 10, i.e., the number of the hives. The training was performed on a GEFORCE RTX 2080 Ti GPU, Intel(R) Core(TM) i7-9700K CPU @ 3.6 GHz, 31 GB RAM, with Ubuntu 22.04.4 LTS. The value of 500 was found experimentally by starting with 50 epochs, incrementing the number of epochs by 50, and observing the performance of trained models on the test data. The performance of the models plateaued at 500, i.e., training for 600, 650, 700, 750, 800, 850, 900, 950, and 1000 epochs did not result in any improvement on the test data. Thus, the value of 500 provided a balance between predictive quality and the computational burden of training the ML models.

To fit the ARIMA models, we replaced each $z_{t}$ in (5) by its form in (4), which makes the forecasting model

(6) $$\begin{array}{ccc} {\hat{x}}_{t} & = & \hat{\delta }+\left(1+{\hat{\phi }}_{1}\right)x_{t-1}+\left({\hat{\phi }}_{2}-{\hat{\phi }}_{1}\right)x_{t-2}+\cdots +\left({\hat{\phi }}_{k-1}-{\hat{\phi }}_{k-2}\right)x_{t-k+1}-{\hat{\phi }}_{k-1}x_{t-k}, \end{array}$$

where we obtained the parameter estimates $\left\{{\hat{\phi }}_{1},{\hat{\phi }}_{2},\ldots ,{\hat{\phi }}_{k-1}\right\}$ and $\hat{\delta }$ with the SAS ARIMA time series fitting procedure. Equation (6) shows how ARIMA uses the *k* previous observations to forecast the next *k* observations and thus matches the model complexity of the three ML models.

We evaluated each fitted ML model on the appropriate test observation sequence with the MSE formula in (3) and plotted its performance against the ground truth values, i.e., the actually observed hourly means. The ARIMA models were also evaluated on the exact same test data for each possible combination of IN and OUT values in Table 4. Specifically, in evaluating ARIMA models, we let OUT =r∈{1,…,k} and indexed the test set observations by t=n+1,…,n+s, where *s* is the number of observations in the test set. Then, for each m∈{n+1,…n+s}, we computed the ARIMA forecasts *r* hours ahead (cf. Equation (6)) for a given fitted ARIMA model as

(7) $$\begin{array}{ccc} {\hat{x}}_{m+r} & = & \hat{\delta }+\left(1+{\hat{\phi }}_{1}\right){\hat{x}}_{m+r-1}+\left({\hat{\phi }}_{2}-{\hat{\phi }}_{1}\right){\hat{x}}_{m+r-2}+\cdots +\left({\hat{\phi }}_{m}-{\hat{\phi }}_{m-1}\right)x_{m}+\ldots \\ \; & \; & +\left({\hat{\phi }}_{k-1}-{\hat{\phi }}_{k-2}\right)x_{m+r-(k-1)}-{\hat{\phi }}_{k-1}x_{m+r-k}, \end{array}$$

where the previous predicted values are used for the first r−1 values of *x* and the remaining values of *x* are the actually observed values when the starting time span is fixed at *m*. For each *m* and each *r*, the predicted value from (7) was subtracted from the actual value, and this difference was squared to obtain ${\left(x_{m+r}-{\hat{x}}_{m+r}\right)}^{2}$. These squared errors were then averaged over the s−k observations in the test set to produce the MSE value for *r* with the MSE formula in (3).

Since the initialization of the ML models in Keras involves the assignment of random weights, we visually inspected each ML plot for topological fitness, i.e., how closely the shape of the curve of the predicted mean values followed the shape of the ground truth curve of the actually observed mean values. For each hive and each ML model, we chose the topologically fit, lowest MSE model and compared it with the ARIMA model for the same hive, the same random variable, and the same span. Topologically unfit models were discarded.

## 4. Results

Due to a very large volume of the experimental results, we have confined most of our tables and figures in this section to hives 2059 (a representative of the Russian queenline) and 2146 (a representative of the Wooten queenline) and to the IN = 24 forecasters for these hives, i.e., the forecasters with the intake of 24 h. Training one ANN model for 500 epochs took ≈7 min. Training one CNN/LSTM model for 500 epochs took ≈10 min. Fitting one ARIMA model took ≈30 s. The plots and tables of the IN = 6 and IN = 12 forecasters for these two hives are given in the document ST.pdf (ST abbreviates *supplementary tables*) ) in the zip archive with the supplementary materials. When we reference a table in ST.pdf, we reference it as **ST X**, e.g., Table **ST 1**. The plots and tables for the other eight hives are given in the supplementary materials or can be easily reconstructed from the CSV files and the trained models therein (cf. README in the supplementary zip). There is no loss of generality in our presentation decision because all univariate forecasters of the same random variables showed the trends and patterns very similar to the trends and patterns of the forecasters for hives 2059 and 2146 discussed in this section and the supplementary document ST.pdf. Tables **ST 1** and **ST 2** in ST.pdf give the results of the most frequent IN = 6 and IN = 12 weight forecaster model types with the minimum MSE for all 10 hives. The top subtable of Table 5 gives the statistics for the IN = 24 weight forecaster model types. Table **ST 1** shows that on IN = 6 (intake of 6 h), the ARIMA forecasters were the most frequent forecasters with the minimum MSE on each predicted horizon with an overall minimum MSE count of 55 out of the 60 possible trained models. On IN = 12, shown in Table **ST 2**, the ARIMA forecasters were also the most frequent minimum MSE forecasters on each predicted horizon, with an overall count of 80 out of the 120 possible trained models. On IN = 24, given in the top subtable of Table 5, ARIMA had 21 minimum MSE forecasters out of the 40 possible trained models. In summary, in forecasting weight, ARIMA outperformed its ML counterparts on all intakes and horizons.

Tables **ST 3** and **ST 4** in ST.pdf give the results of the most frequent IN = 16 and IN = 12 in-hive temperature forecaster model types with the minimum MSE for all 10 hives. The middle subtable in Table 5 gives these statistics for IN = 24 in-hive temperature forecasters. Table **ST 3** shows that on IN = 6, LSTM was the most frequent model with the minimum MSE: 46 LSTM forecasters had the minimum MSE out of the 60 possible trained models. ARIMA came in second with the minimum MSE count of 12. On IN = 12, shown in Table **ST 4**, LSTM was again the most frequent minimum MSE model: 71 minimum MSE forecasters out of the 120 possible trained models. On IN = 24, shown in the middle subtable in Table 5, ARIMA was the most frequent minimum MSE model type, with 35 minimum MSE forecasters out of the 40 possible trained models. In summary, on IN = 6 and IN = 12, in forecasting in-hive temperature, LSTM was the top forecaster model type with ARIMA coming second; on IN = 24, ARIMA outperformed its ML counterparts.

Tables **ST 5** and **ST 6** in ST.pdf give the results of the most frequent IN = 16 and IN = 12 traffic forecaster model types with the minimum MSE for all 10 hives. The bottom subtable in Table 5 gives these statistics for IN = 24 traffic forecasters for all 10 hives. In all three tables, the traffic counts were computed with OmniBeeM with a YOLOv3 bee object inference model. On IN = 6, shown in Table S5, LSTM was the most frequent minimum MSE model type, with 44 minimum MSE forecasters out of the 60 possible trained models. ANN and CNN shared the second place with 7 minimum MSE forecasters. ARIMA had 2 minimum MSE forecasters. On IN = 12, given in Table **ST 6**, ANN had the largest count of minimum MSE forecasters with 101 forecasters, out of the 120 possible trained forecasters. CNN came second with 10 forecasters; LSTM—third with 7 forecasters, and ARIMA—fourth with 2 forecasters. On IN = 24 in the bottom subtable in Table 5, ANN was the most frequent minimum MSE model: 32 minimum MSE forecasters out of the 40 possible trained models; ARIMA was second with 8 forecasters. Neither CNN nor LSTM had any minimum MSE forecasters. In summary, on IN = 6, LSTM was first, ANN and CNN second, and ARIMA third; on IN = 12, ANN was first, CNN—second, LSTM—third, and ARIMA—fourth; on IN = 24, ANN was first, ARIMA—second, and CNN and LSTM did not have any minimum MSE forecasters.

Table **ST 7** in ST.pdf gives the minimum MSE plots for the best IN = 6 and IN = 12 weight forecasters for hives 2056 and 2146. For hive 2056 on IN = 6, the minimum MSE ranged from 0.0002 to 0.0016, with an increase in MSE on longer horizons. For hive 2059, on IN = 12, the minimum MSE ranged from 0.0001 to 0.003, with an increase in MSE on longer horizons. For hive 2146, on IN = 12, the minimum MSE ranged from 0.00001 to 0.0061. Row 1 in Table 6 gives the plots of the minimum MSE values of the best IN = 24 weight forecasters for hives 2059 and 2146. For hive 2059, on IN = 24, the minimum MSE ranged from 0.001 to 0.008, with an overall increase in MSE on longer horizons. For hive 2146, on IN = 24, the minimum MSE ranged from 0.0015 to 0.004. ANN, CNN, and ARIMA had an overall increase in MSE on longer horizons. LSTM showed a parabolic shape with the MSEs on the horizons of 6 and 24 slightly higher than on the horizons of 12 and 18. In summary, the small MSE ranges on all intakes and horizons indicate that the weight forecasters differed only slightly in terms of the predictive power on all time spans. On all spans, in forecasting hive weight, ARIMA performed on par with its ML counterparts.

Table **ST 8** in ST.pdf gives the minimum MSE plots for the best IN = 6 and IN = 12 temperature forecasters for hives 2056 and 2146. For hive 2056, on IN = 6, the minimum MSE ranged from 0.001 to 0.175 with an increase in MSE on longer horizons, with the exception of LSTM on the horizon of 3 h (OUT = 3) where its MSE was higher than the MSEs of the other three models. For hive 2146, on IN = 6, the minimum MSE ranged from 0.01 to 0.07 with an increase in MSE on longer horizons, with the exception of LSTM on the horizon of 3 h (OUT = 3) where its MSE was higher than the MSE of the other three models. For hive 2059, on IN = 12, the minimum MSE ranged from 0.01 to 0.10, with all models showing an overall MSE on longer horizons. LSTM had a higher MSE on the horizon of 6 than the other three forecasters. For hive 2145, on IN = 12, the minimum MSE ranged from 0.01 to 0.062, with all models MSE curves showing the inverse parabolic shape, i.e., the middle horizons having slightly higher MSEs than the end horizons of 1 and 12. Row 2 in Table 6 gives the MSE plots for the best IN = 24 temperature forecasters for hives 2059 and 2146. On this intake, the MSE ranged from 0.03 to 0.05 for hive 2059, with the MSE rising on the longer horizons for CNN and LSTM and being inversly parabolic for ANN and ARIMA. For hive 2146, the MSE ranged from 0.03 to 0.0475, with a falling MSE curve for LSTM, a rising curve for CNN, an inversely parabolic curve for ANN, and a flat curve for ARIMA. In summary, on hives, some temperature forecasters exhibited inversely parabolic MSE. In forecasting temperature, ARIMA performed on par with its ML counterparts and outperformed them on the longer horizons for hive 2059 and on all horizons for hive 2146. The small MSE ranges on all intakes and horizons of the temperature forecasters indicate that the in-hive temperature forecasters differed very slightly in terms of the predictive power on all time spans.

Table **ST 9** in ST.pdf gives the minimum MSE plots for the best IN = 6 and IN = 12 traffic forecasters for hives 2056 and 2146. In predicting the traffic computed with OmniBeeM with our trained YOLOv3 flying bee object inference model. For hive 2059, on IN = 6, the minimum root MSE (RMSE) varied from 192 bees to 252 bees for hive 2059 and from 600 bee objects to 1410 bees for hive 2146. The MSE plots for hive 2059 showed an overall increase in MSE for longer horizons. For hive 2146, all plots except ARIMA’s had inversely parabolic shapes. For hive 2059 on IN = 12, the RMSE from 196 bees to 235 bees. For hive 2146, on IN = 12, the RMSE varied from 610 bees to 1205 bees. The RMSE of LSTM and CNN jumped up and down for hive 2059. The RMSE of LSTM was varied on different horizons. For both hives, on IN = 6 and IN = 12, ANN turned out to be the most stable forecaster with the smallest RMSE. Row 3 in Table 6 gives the RMSE plots for the best IN = 24 traffic forecasters for hives 2059 and 2146. For hive 2059 on this intake, RMSE ranged from 180 bees to 215 bees; for hive 2146 on the same intake, RMSE ranged from 600 bees to 1180 bees. For hive 2059, ANN, CNN, and ARIMA forecasters had inversely parabolic shapes, and LSTM’s RMSE fluctuated up and down. ANN showed the smallest RMSE on all horizons. On the longer horizons of 12, 18, and 24 h, ARIMA had the second-smallest RMSE. In particular, the difference between ANN and ARIMA at 18 and 24 h and the RMSE differences between ANN and ARIMA were less than 15 bees. For hive 2146, ANN had the smallest RMSE on all horizons. ARIMA had the second-smallest RMSE on all horizons. CNN had the third-smallest RMSE on all horizons. LSTM’s RMSE jumped up and down from horizon to horizon. On the intakes of 6 and 12 h, the RMSE of ANN and ARIMA were basically identical and did not exceed 650 bees. On the longer intakes of 18 and 24 h, the RMSE of ANN and ARIMA slightly increased but differed by approximately 50 bees.

While the RMSE of the traffic forecasters may appear to be significantly different, in reality, it is not the case when we take into consideration the fact that on many days the hourly counts of flying bees at the hive’s entrance of a healthy colony with two boxes are in thousands. Thus, e.g., the difference of fewer than 15 bees between the best IN = 24, OUT = 24 ANN forecaster and the second best IN = 24, OUT = 24 ARIMA forecaster for hive 2059 (cf. left plot in Row 3, Table 6) is not significant.

Tables **ST 10**–**ST 21** in ST.pdf show the plots of the predictions of all 6–1 (IN = 6, OUT = 1), 6–6 (IN = 6, OUT = 6), 12–1 (IN = 12, OUT = 1), and 12–12 (IN = 12, OUT = 12) forecasters weight, temperature, and traffic forecasters for hives 2059 and 2146. Table 7 and Table 8 in this section show the plots of the predictions of all IN = 24, OUT = 6, and IN = 24, OUT = 24 forecasters for hives 2059 and 2146, respectively. These plots illustrate that all forecasters of the three random variables predict long-term trends in the corresponding time series equally well. While the individual predictions of the best forecasters may differ from the actually observed values, the predicted and observed curves remained topologically close to each other.

To support the previous observation with more evidence, we included Table 9 with the plots of the predictions of all IN = 24, OUT = 6, and IN = 24, OUT = 24 forecasters for hives 2123. These plots indicate that all forecaters can predict not only the falling or flat trends in the test data, as is the case with hives 2059 and 2146, but also the rising and falling trends, as is the case with hive 2123.

## 5. Discussion

A key aim of our investigation was to assess the relative utility of three frequently used ML models (i.e., ANN, CNN, and LSTM) for predicting univariate time series and to benchmark them against the traditional ARIMA time series methodology, using three highly distinct, in their statistical properties, measures of hive health (i.e., hive weight, in-hive temperature, and hive entrance traffic). Since all four univariate forecaster models performed on par in terms of MSE and trend prediction, the actual choice of a univariate forecaster for hive weight, in-hive temperature, or hive entrance traffic may have to be guided by other considerations. For example, if continuous access to cloud computing is assured for a hive monitoring system, which, in turn, makes possible the continuous training of forecaster models on growing data quantities, then the results of our investigation suggest that it does not matter which model is chosen insomuch as all models will likely capture trends in the time series that they are trained to predict. However, if cloud access is not available or affordable, then ARIMA should be chosen because ARIMA models, unlike their ML/DL counterparts like ANNs, CNNs, and LSTMs, do not require GPU computers, which has an important practical implication for apiary science researchers interested in deploying embedded systems that can monitor managed colonies in situ.

Due to smaller memory footprints, ARIMA models can be fitted to large datasets in a matter of minutes even on restricted, embedded platforms such as the Raspberry Pi platform that we used in this investigation for video data collection, which has important implications for multisensor hive monitoring systems because embedded platforms have smaller energy footprints than GPU farms or cloud computer clusters (cf., e.g., Kulyukin and Kulyukin, 2023 [40]). Incidentally, our focus on shallow ANN, CNN, and LSTM models was, in fact, motivated by the fact that deeper ML models have larger memory footprints, which makes it hard or impossible to run them in situ on embedded platforms (cf., e.g., Kulyukin et al., 2021 [44]).

Another consideration for apiary science researchers and practitioners is that training the ML models was more computationally expensive relative to ARIMA fitting; e.g., it took, on average, ≈7 to 10 min of computational time to train one ML model for 500 epochs, whereas the ARIMA fit to the training dataset took less than a minute. However, once the training was completed, computing the predicted MSE on the test set took 1–2 s for all models.

Since all four methods performed on par when evaluated by the MSEs computed on the test datasets, a theoretical implication for continuous hive monitoring is that natural cycles of managed colonies might be mathematically described and, consequently, predicted with various degrees of accuracy. In particular, when used as DUTS forecasters, ANNs, CNNs, and LSTMs can be construed as applying a sliding filter of a specific length over the value of the time series. These filters have only one dimension (i.e., time) instead of 2 or more dimensions (i.e., width, height, transparency, red, blue, green channels, etc., as is the case of images). In other words, these filters are non-linear transformations of a given time series. For example, an IN = 12, OUT = 12 model runs a filter of length 12 on a DUTS and applies a moving average with a sliding window of length 12.

The potential for over- or underfitting always exists and can never be completely eliminated. We addressed this weakness by using the different model complexities (e.g., IN = 6, OUT = 6; IN = 12, OUT = 12; IN = 24, OUT = 24). Our results indicate that the least complex IN = 6, OUT = 6 models (i.e., the models with the potential to underfit, because they involved only 6 input and 6 output parameters), the middle complexity IN = 12, OUT = 12 models with the potential to underfit or overfit, and the most complex IN = 24, OUT = 24 models (i.e., the models most likely to overfit, because they had 24 input and 24 output parameters) had similar performance on the test data. Furthermore, overfitting was unlikely to be present since each model’s complexity and performance were assessed only on the test data of each DUTS with a 70/30 train/test split.

As electronic sensors become mainstream and smaller in size, we expect that more of them will be integrated into precision apiculture systems capable of univariate and multivariate predictive modeling. It is unlikely, however, that a single sensor will be sufficient to characterize or predict the behavior or status of a managed bee colony completely. Rather, future predictive models for precision apiculture will likely rely on multiple sensors, such as scales, in-hive temperature sensors, and video sensors. It is impossible to predict at this point whether some or all of these sensor types will be used in future multisensor systems because each of the three sensor types has its own relative advantages and disadvantages, which we now proceed to review briefly on the basis of our longitudinal experiences with them.

The popularity of electronic hive scales among apiary scientists may be due to their availability, non-invasiveness, ergonomics, and relevance (Kulyukin et al., 2022 [39]). Placing hives on scales does not interfere with the natural cycles of managed colonies. The scales do not require any structural modifications of the hive and provide a continuous, interpretable hive-specific data stream around the clock regardless of ambient weather conditions. The scales are ergonomic because, while they may be awkward to move, they do not interfere with hive inspections, and relevant because they provide data that bears on the status and productivity of the hive. However, the scales are relatively hard to move from apiary to apiary or within a large apiary and may require initial calibration and subsequent repeated recalibration. If the initial calibration or a scheduled recalibration is skipped or not properly carried out, the quality of data may be compromised.

Like scales, in-hive temperature sensors are non-invasive and relevant. An advantage of temperature sensors over scales is their small size and smaller power footprints, which makes them more mobile and more easily deployable than scales. However, unlike external scales, temperature sensors may be subject to destruction when the hives in which they are placed are infected. For example, in Arizona and Utah, the hives infected with the American Foul Brood must be promptly destroyed by the agricultural authorities. In some situations, depending on the degree and type of colony infection, temperature sensors may be recovered from infected hives by being treated with alcohol or some other appropriate disinfectant due to their small size. Since most temperature sensors are small and attached to frames, they can be lost during inspections or when hives are moved from apiary to apiary or within a large apiary. The reviewed literature shows that in-hive temperature is relevant because it bears on the status of the colony. However, interpreting it may be more challenging than interpreting hive weight or hive entrance traffic. For example, the data extracted from an in-hive temperature sensor is likely to depend on where in the hive it is placed (cf., e.g., Szabo et al., 1989 [23]; Meikle et al., 2016 [26]).

Video cameras also have their relative advantages and disadvantages. They are easy to deploy, ergonomic, and relevant, insomuch as, when used externally, they are not subject to destruction due to hive infections or to loss during hive movement. Hive entrance traffic is also relevant to the status of the colony. However, the video quality is dependent on ambient weather, unless the lighting conditions are controlled for, which adds to the cost and complexity. Furthermore, if hive entrance traffic quantification is performed, in part, with ML or DL models, the resultant methods may not transfer from location to location or from bee race to bee race due to biases in the training data that may be hard to detect or eliminate.

A broader context for our investigation is the use of DUTS in-hive weight, in-hive temperature, and hive entrance traffic forecasters in the longitudinal monitoring of different queenlines in order to compare the colony-level behaviors of flight activity, foraging effort and success, and thermoregulation. This is important in order to understand the relative advantages and disadvantages of some bee stocks in commercial applications such as pollination and honey production. The two bee stocks investigated in this study by the entomological members of our research team (Meikle and Weiss) have so far been found to differ in terms of factors such as worker survivorship, cluster temperature, and food consumption in laboratory studies (Meikle et al., 2024 [45]). However, we are far from understanding how these factors manifest themselves in managed colonies and, consequently, how we can use them in predictive models. Since the choice of appropriate bee stocks can represent a considerable investment (e.g., purchase of queens and bee packages) for commercial beekeepers, an improved understanding of colony-level behaviors is important for the entire industry, including queen breeders.

It is also important to note that our methods may appeal to apiary science researchers interested in natural colony cycles because we are investigating *non-invasive* predictive modeling; we aim to construct predictive models by using only the sensors that do not interfere with the natural cycles of managed colonies. For instance, Kulyukin and Mukherjee (2019) [43] formulated the principle of non-invasiveness in precision apiculture as follows:

“… the sacredness of the bee space must be preserved in that the deployment of EBM [electronic beehive monitoring] sensors should not be disruptive to natural beehive cycles.”

## 6. Conclusions and Future Work

A principal conclusion of the first part of our investigation is that the mean hive weight, in-hive temperature, and hive entrance traffic of all 10 colonies could be predicted with a reasonable degree of accuracy on the time spans of 12, 24, and 48 h. Another conclusion is that, on the curated dataset, the ARIMA forecasters performed on par with the ANN, CNN, and LSTM forecasters. Since ARIMA models can be fitted to large datasets rather quickly on hardware platforms with smaller memory footprints, they may be a reasonable alternative to more sophisticated ML and DL models if wireless access to cloud computing services or to local GPU farms is not available or not affordable. Running trained ML models on embedded platforms in situ can be challenging because their RAM memory footprints may be prohibitively large for real-time deployment or execution. Our findings and conclusions about the DUTS of hive weight, in-hive temperature, and hive entrance traffic should be interpreted only in the context of the data from the ten managed hives in Tucson, AZ, USA, and may not generalize to other climates or bee races.

In Part II of our investigation, which we intend to cover in the next article, we plan to investigate relative differences in the traffic forecasters when traffic is measured by OmniBeeM coupled not only with YOLOv3, but also with YOLOv4-Tiny and YOLOv7-Tiny. We have quantified all captured videos with traffic measurements obtained with these three trained bee object inference models but have not yet trained all possible DUTS forecaster model types on the YOLOv4-Tiny and YOLOv7-Tiny traffic data or analyzed their relative performance on the test data. We also plan to investigate the feasibility of learning transfer, i.e., how well the forecaster models trained on one hive’s data can predict the test data from a different hive. If the answer to this question turns out to be positive, then we can entertain the possibility of constructing queenline-independent forecasters. If, on the other hand, the answer turns out to be negative, then we can hypothesize that the hive weight, in-hive temperature, and hive entrance traffic may be colony-specific and design studies to investigate this hypothesis.

## Figures and Tables

**Table 1:** **Total numbers of logged weight and temperature readings and videos.** The weight data were logged every 5 min around the clock from 00:00 on 18 June 2022 to 00:00 on 23 September 2022 for each hive. The temperature data were logged every 5 min around the clock from 00:00 on 21 June 2022 to 11 October 2022 for each hive. A 30 s MP4 video was recorded by each vlogger every 5 min from 7:00 to 20:55 daily during the video capture periods in the **VCP** column. Table legend: **HID**—Hive ID; **GQL**—Genetic Queen Line; **WRC**—number of hive weight readings captured; **TRC**—number of in-hive temperature readings captured; **VDC**—number of on-hive videos captured; **TOT**—total number of records captured; **VCP**—video capture period.

HID	GQL	WRC	TRC	VDC	TOT	VCP
2059	Russian	28,076	32,257	18,263	78,596	11:05,06/24–20:55,10/10
2120	Russian	28,076	32,257	18,281	78,614	12:00,06/24–09:20,10/11
2123	Wooten	28,076	32,257	16,575	76,908	10:35,06/24–20:55,09/30
2129	Wooten	28,076	32,257	17,056	77,389	13:40,06/24–20:55,10/03
2130	Wooten	28,076	32,257	16,551	76,884	13:45,06/24–20:55,09/30
2137	Wooten	28,076	32,257	18,293	78,626	11:40,06/24–10:00,10/11
2141	Russian	28,076	32,257	4623	64,956	13:45,06/24–20:55,07/21
2142	Russian	28,076	32,257	18,270	78,603	13:40,06/24–10:05,10/11
2146	Wooten	28,076	32,257	18,231	78,564	13:45,06/24–20:55,10/10
2158	Russian	28,076	32,257	9230	69,563	13:40,06/24–12:45,08/18
**TOT**		280,760	322,570	155,373	758,703	

**Table 2:** **Weight, temperature, and traffic means for hour 10 on 7 July 2022.** The means for hour 10 are computed from the 12 weight, temperature, and traffic measurements from 9:00 up to 9:55. The mantissas of real numbers are rounded to 2 digits. Table legend: HID—Hive ID; HR—hour (a non-negative integer used instead of a time stamp); μW—mean hive weight (kg) for hour 10; μC—mean in-hive temperature (degrees Celsius) for hour 10; μY3—mean omnidirectional bee motion count obtained with OmniBeeM with YOLOv3 for hour 10; μY4T—mean omnidirectional bee motion count obtained with OmniBeeM with YOLOv4-Tiny for hour 10; μY7T—mean omnidirectional bee motion count obtained with OmniBeeM with YOLOv7-Tiny for hour 10.

HID	HR	μW	μC	μY3	μY4T	μY7T
2059	10	15.70	35.38	226.42	1300.33	610.42
2120	10	15.76	35.93	4132.50	941.42	802.42
2129	10	14.79	35.89	1666.33	349.83	493.83
2123	10	14.84	35.16	930.42	338.42	507.92
2130	10	14.04	35.36	926.17	403.17	435.42
2137	10	16.41	35.38	1975.58	1017.92	556.58
2141	10	13.55	35.12	1051.67	95.17	218.00
2142	10	13.74	35.22	1705.67	856.00	460.67
2146	10	16.01	35.48	3319.33	1340.58	1184.00
2158	10	16.50	35.58	420.08	455.08	381.83

**Table 3:** **Total numbers of weight, temperature, and traffic means used in time series forecasting.** Table legend: HID—Hive ID; NWM—number of hourly hive weight means; NCM—number of hourly in-hive temperature means; NBM—number of hourly bee entrance traffic means.

HID	2059	2120	2123	2129	2130	2137	2141	2142	2146	2158
NWM	2160	2160	2160	2160	2160	2160	2160	2160	2160	2160
NCM	2160	2160	2160	2160	2160	2160	2160	2160	2160	2160
NBM	1170	1170	1170	1170	1170	1170	352	1170	1170	708
TOT	5490	5490	5490	5490	5490	5490	4672	5490	5490	5028

**Table 4:** **Forecaster model intake and horizon values.** Table legend: IN—the forecaster’s intake, i.e., the number of input (actually observed) values; OUT—the forecaster’s horizon, i.e., the number of predicted values; the integers in the IN and OUT columns denote numbers of hours; NF—the number of the forecasters with the corresponding values of IN and OUT, i.e., NF = IN × |OUT| × 4, where |OUT| is the cardinality of the set OUT in each row and 4 is the number of the model types, i.e., ANN, CNN, LSTM, and ARIMA. TOT is the total number of forecasters of each model type evaluated in this investigation.

IN	OUT	NF	TOT
6	{ 1, 2, 3, 4, 5, 6 }	6 × 6 × 4	144
12	{ 1, 2, 3, 4, 5, 6, 7, 8, 9, 10, 11, 12 }	12 × 12 × 4	576
24	{ 6, 12, 18, 24 }	24 × 4 × 4	384
			1104

**Table 5:** **IN = 24 minimum MSE forecaster model counts.** Counts of times when a IN = 24 forecaster model trained on the train data (70%) of a hive had minimum MSE on the test data (30%) of the same hive. The counts are reported for all 10 hives. Highest total counts are bolded. For the intake of n=24, the maximum possible total count is 40, i.e., 4 possible IN, OUT pairs for each of the 10 hives (cf. Table 4). For instance, for the weight forecasters, the total ARIMA count of 21 is interpreted as follows: out of 40 models trained and tested on each hive, 21 ARIMA models had the lowest MSE on the test data. The total counts for the temperature and traffic models are interpolated analogously.

	IN	OUT	ANN	CNN	LSTM	ARIMA
	24	6	2	1	0	7
	24	12	2	3	1	4
Weight Forecasters	24	18	3	0	2	5
	24	24	1	0	4	5
	TOT		8	4	7	**21**
	IN	OUT	ANN	CNN	LSTM	ARIMA
	24	6	1	0	1	8
Temp Forecasters	24	12	1	0	1	8
	24	18	0	0	0	10
	24	24	1	0	0	9
	TOT		3	0	2	**35**
	IN	OUT	ANN	CNN	LSTM	ARIMA
	24	6	9	0	0	1
Traffic Forecasters	24	12	8	0	0	2
	24	18	8	0	0	2
	24	24	7	0	0	3
	TOT		**32**	0	0	8

**Table 6:** **Minimum MSE plots for IN = 24 forecasters.** Minimum MSE plots of the best ANN, CNN, LSTM, and ARIMA IN = 24 forecasters on the test data for hives 2059 and 2146. The PDF may have to be enlarged to see the plots. The x-axis in each plot, labeled OUT, is interpreted as follows: 1.0 denotes OUT = 6, i.e., the prediction horizon of 6 hourly means; 2.0 denotes OUT = 12, i.e., the prediction horizon of 12 hourly means; 3.0 denotes OUT=18, i.e., the prediction horizon of 18 hourly means; 4.0 denotes OUT = 24, i.e., the prediction horizon of 24 hourly means.

**Weight Forecasters**	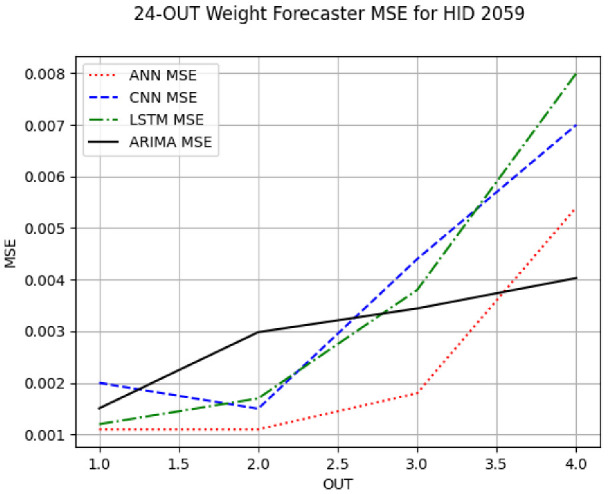 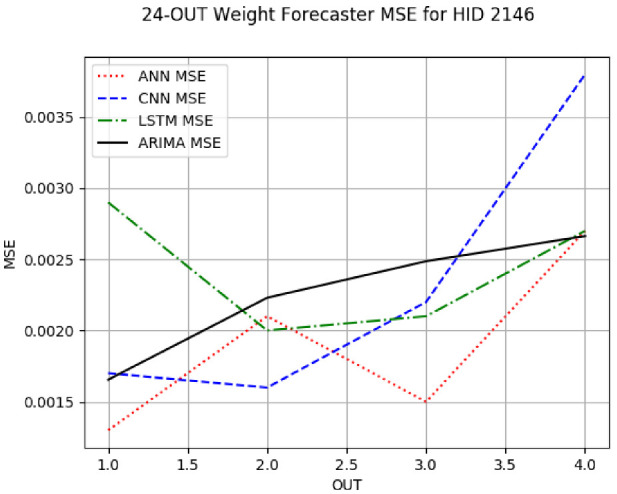
**Temp. Forecasters**	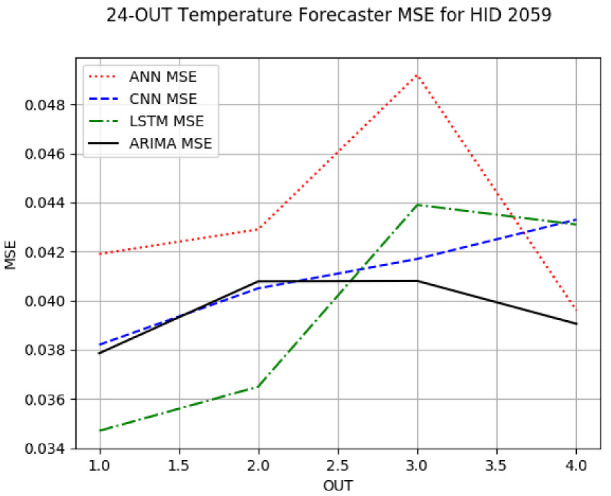 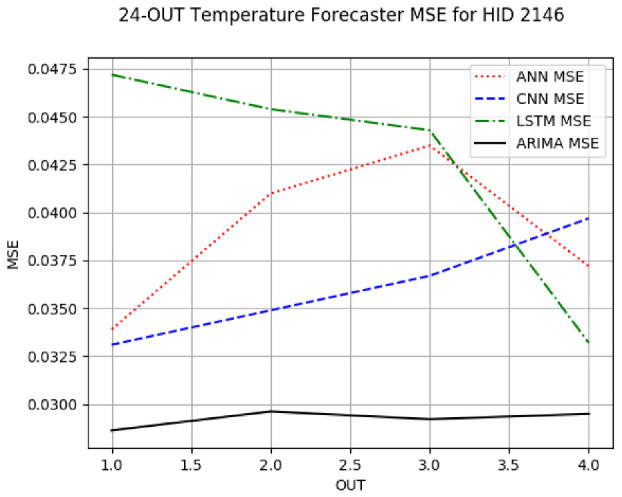
**Traffic Forecasters**	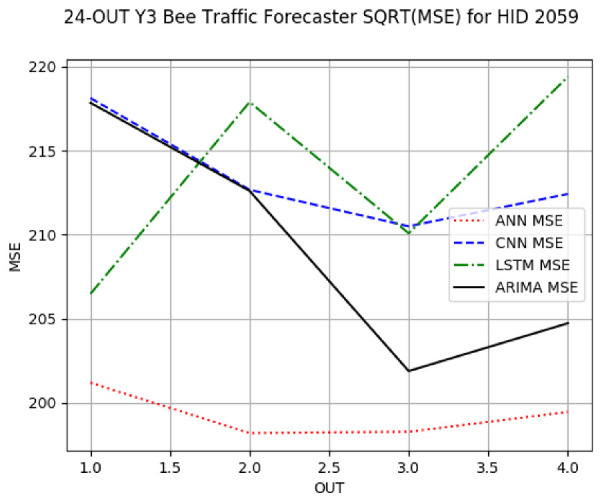 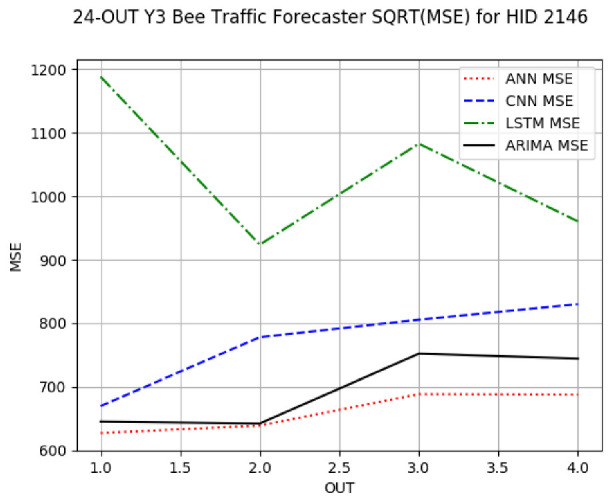

**Table 7:** **Observed vs. predicted MSE plots for IN = 24, OUT = 6, and IN = 24, OUT = 24 forecasters for hive 2059.** The PDF may have to be enlarged to see the plots.

	ANN	CNN	LSTM	ARIMA
**Weight 24-6**	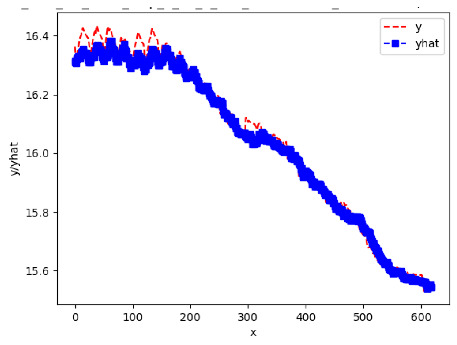	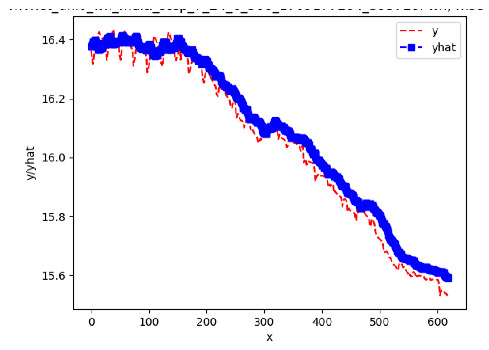	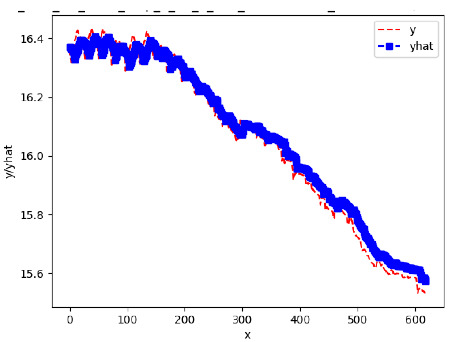	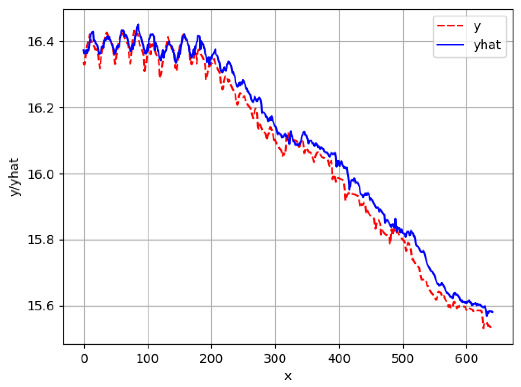
**Weight 24-24**	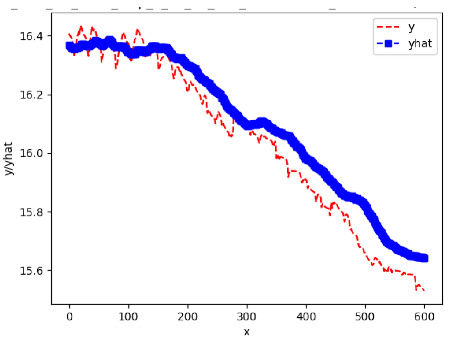	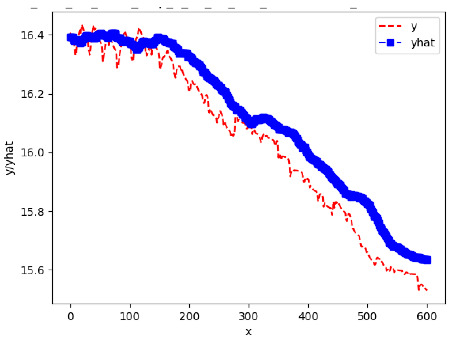	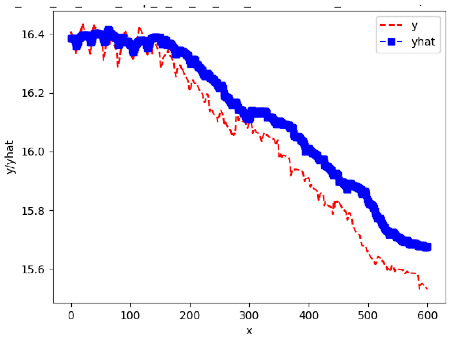	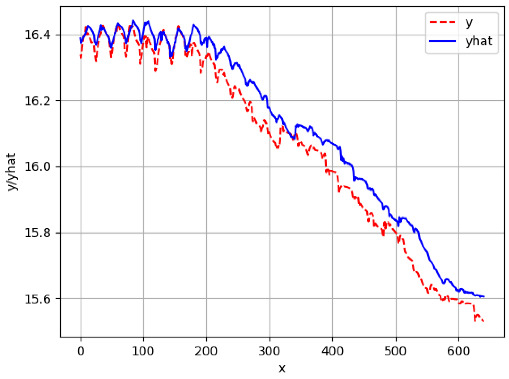
**Temp 24-6**	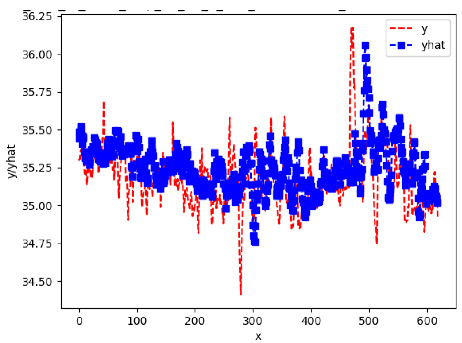	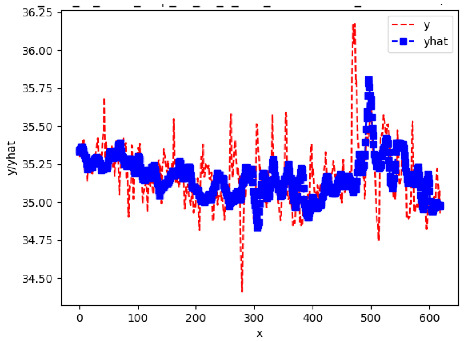	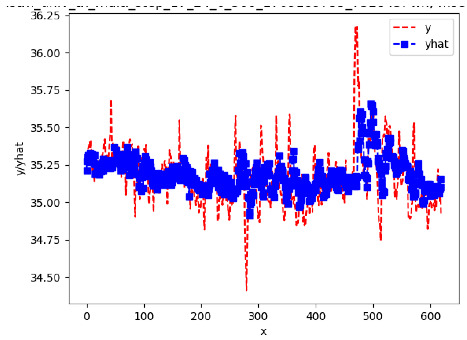	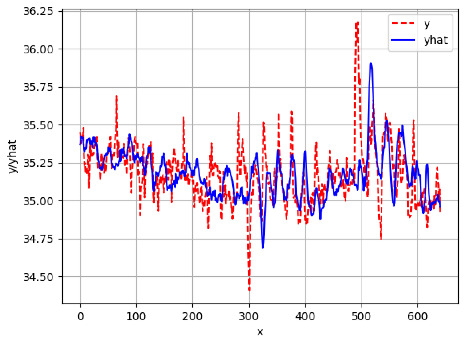
**Temp 24-24**	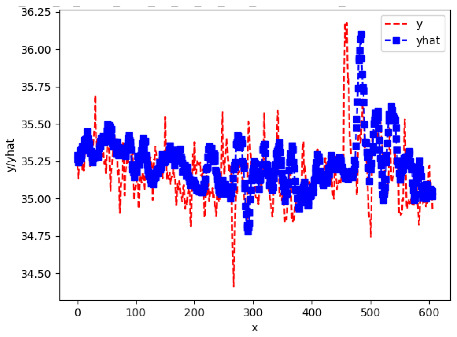	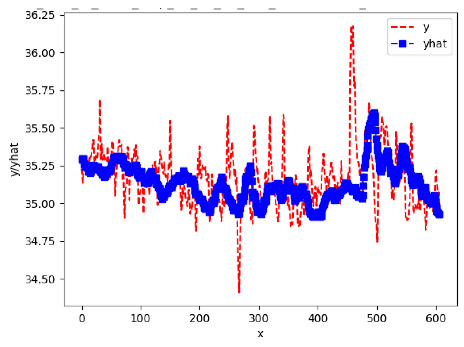	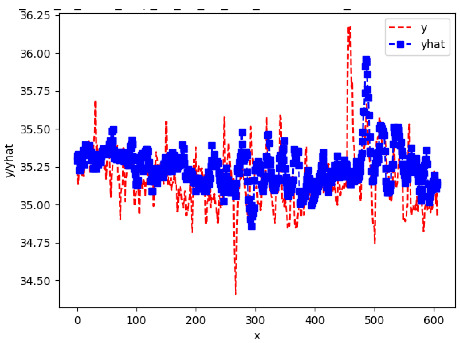	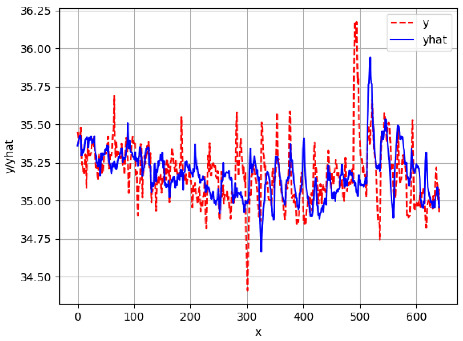
**Traffic 24-6**	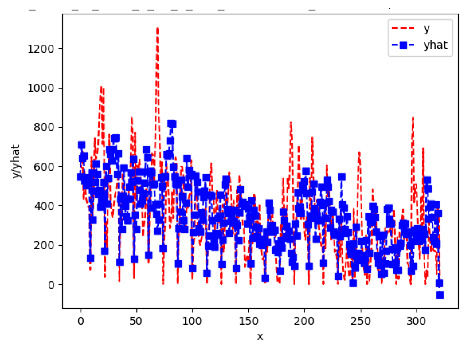	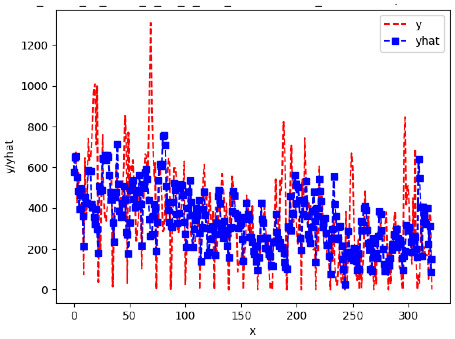	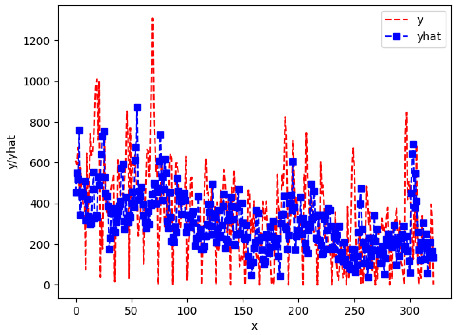	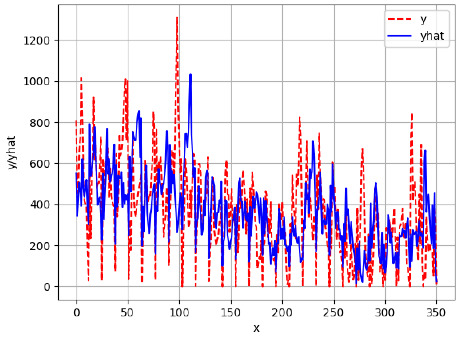
**Traffic 24-24**	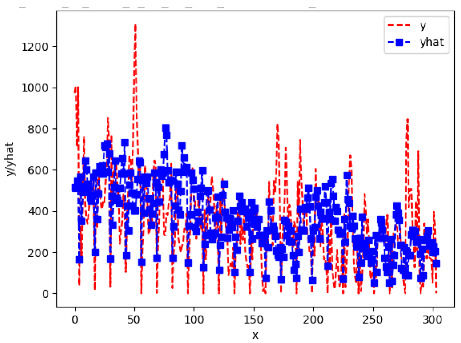	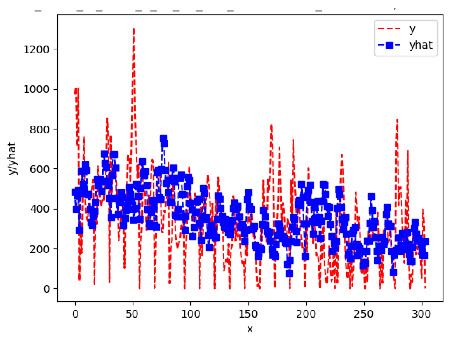	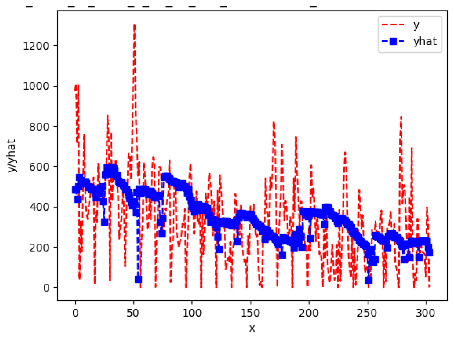	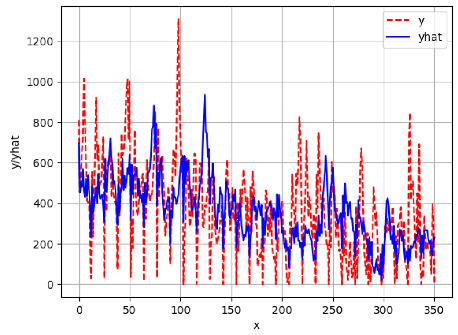

**Table 8:** **Observed vs. predicted MSE plots for IN = 24, OUT = 6, and IN = 24, OUT = 24 forecasters for hive 2146.** The PDF may have to be enlarged to see the plots.

	ANN	CNN	LSTM	ARIMA
**Weight 24-6**	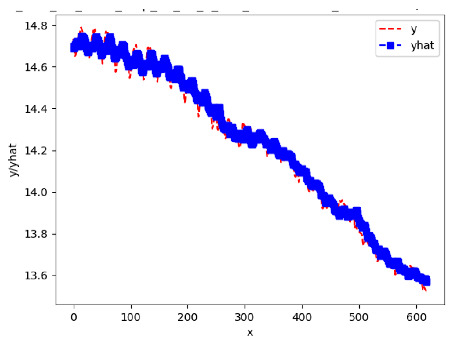	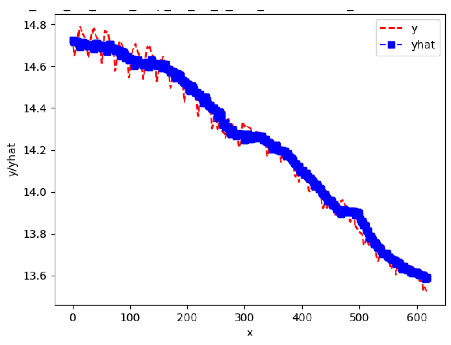	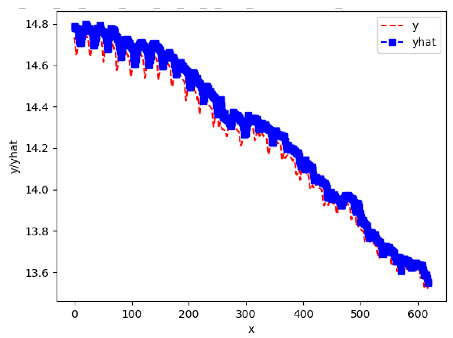	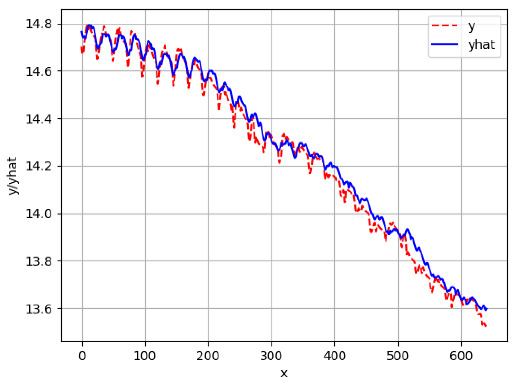
**Weight 24-24**	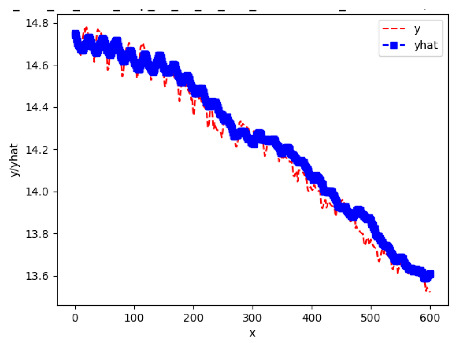	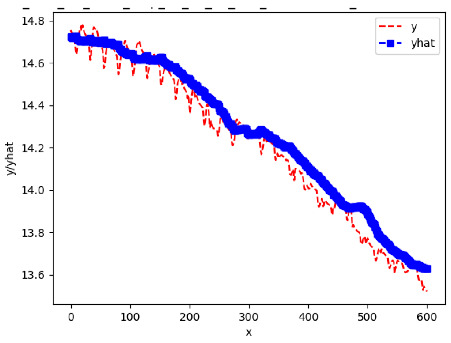	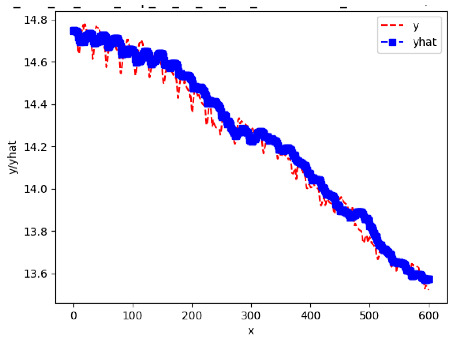	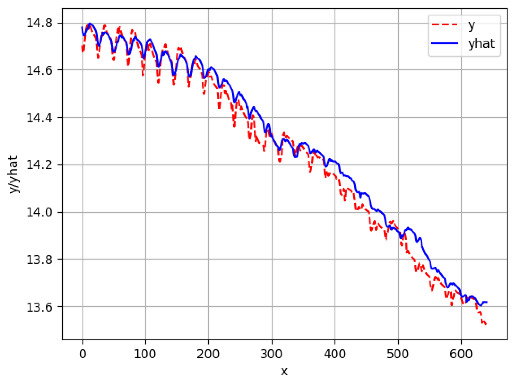
**Temp 24-6**	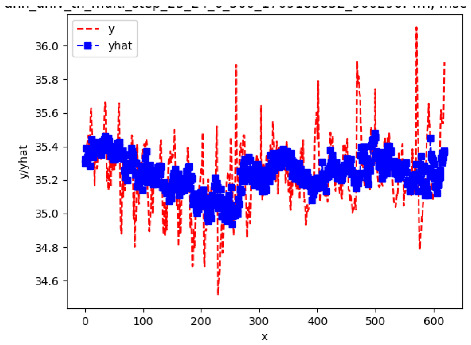	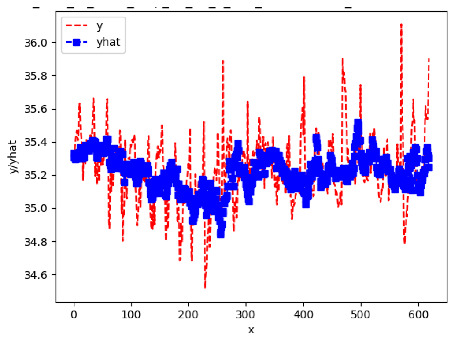	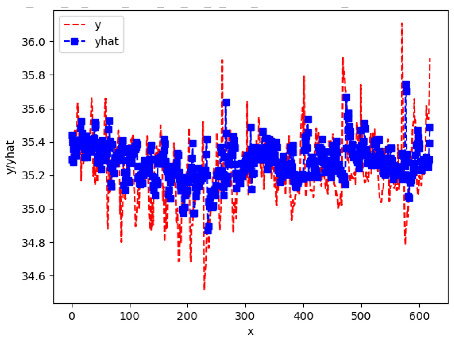	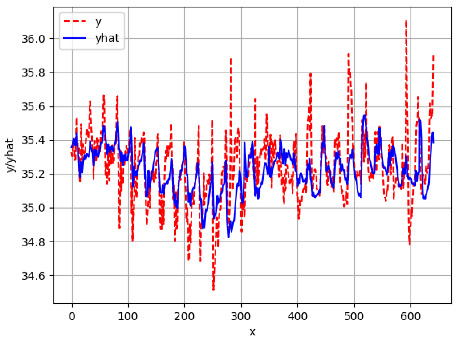
**Temp 24-24**	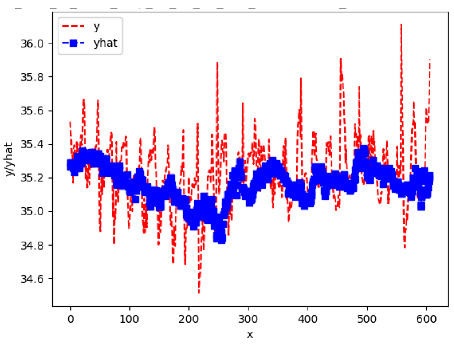	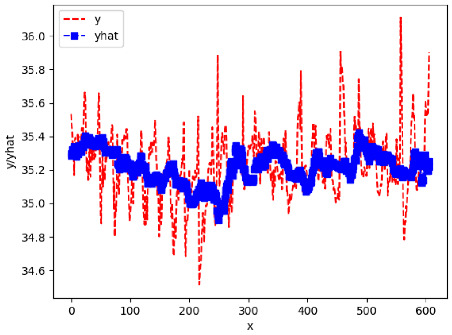	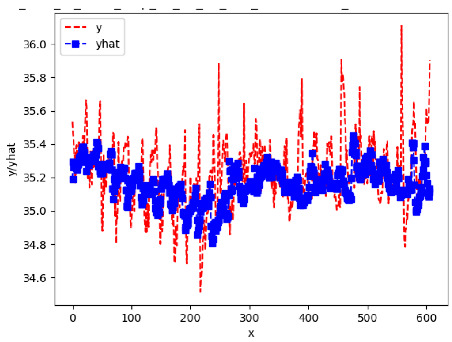	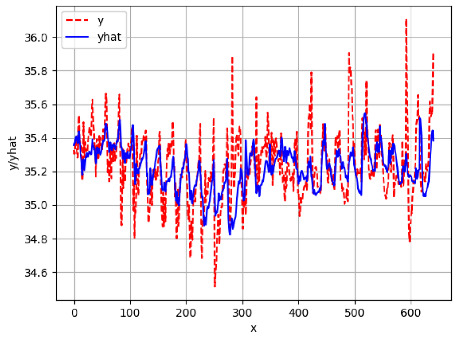
**Traffic 24-24**	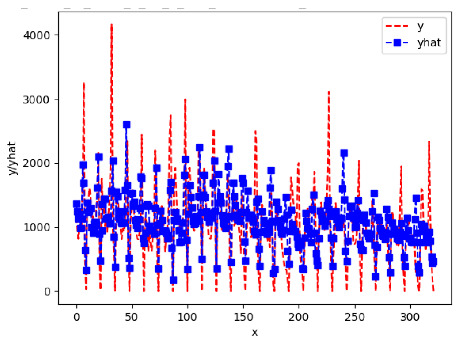	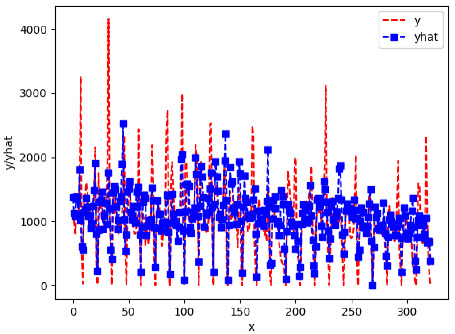	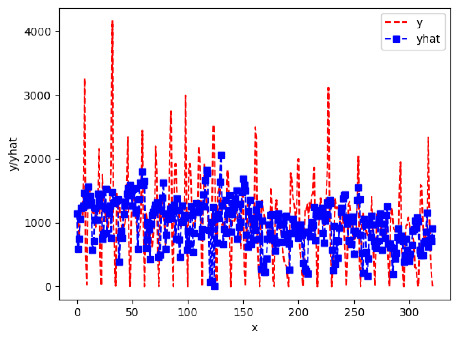	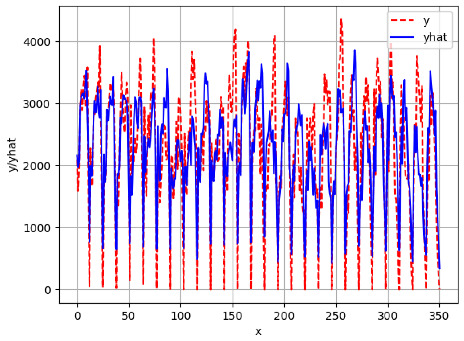
**Traffic 24-24**	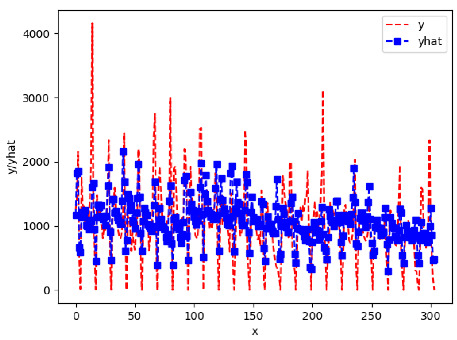	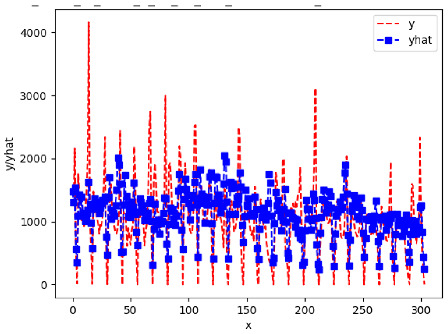	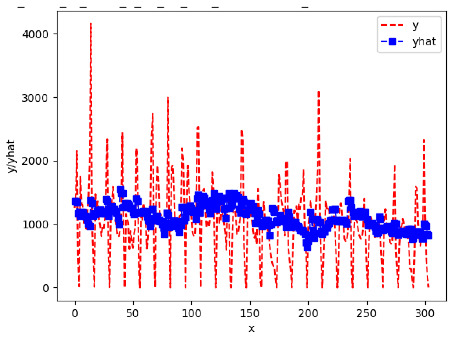	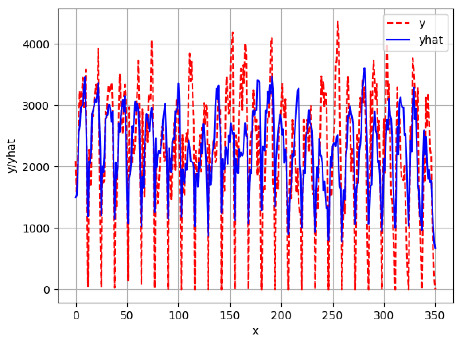

**Table 9:** **Observed vs. predicted MSE plots for IN = 24, OUT = 6, and IN = 24, OUT = 24 forecasters for hive 2123.** The PDF may have to be enlarged to see the plots.

	ANN	CNN	LSTM	ARIMA
**Weight 24-6**	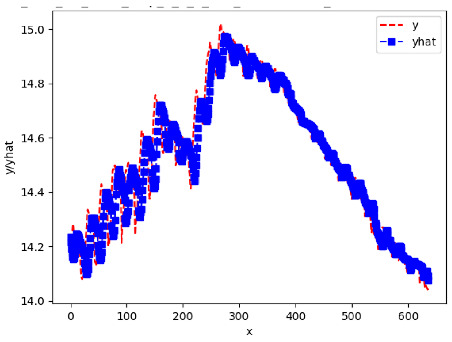	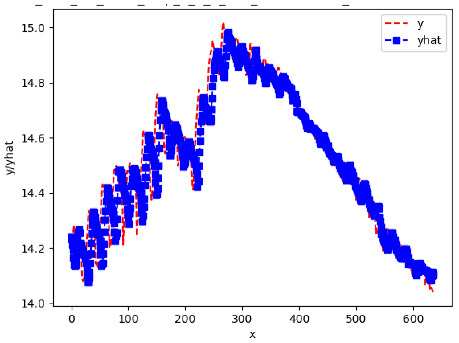	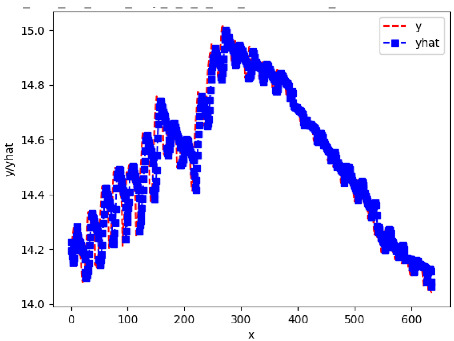	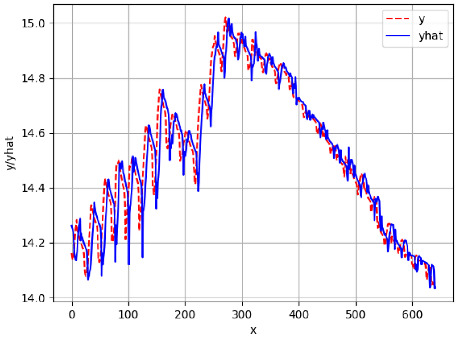
**Weight 24-24**	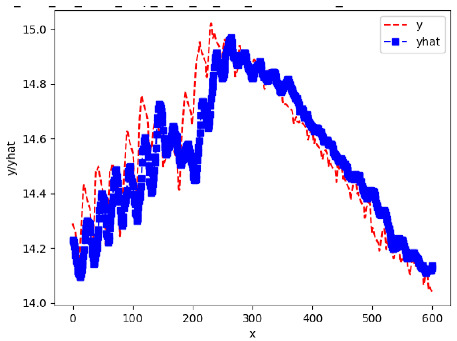	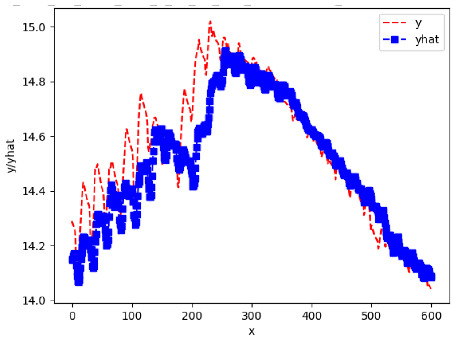	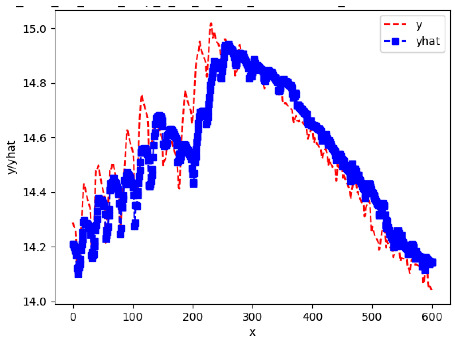	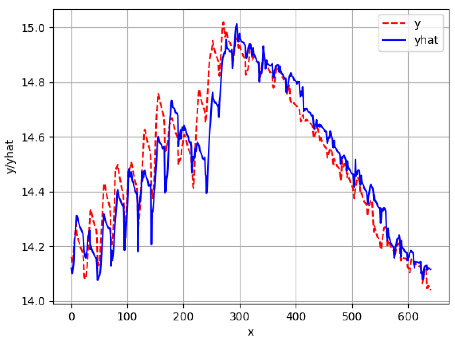

**Figure A2:**
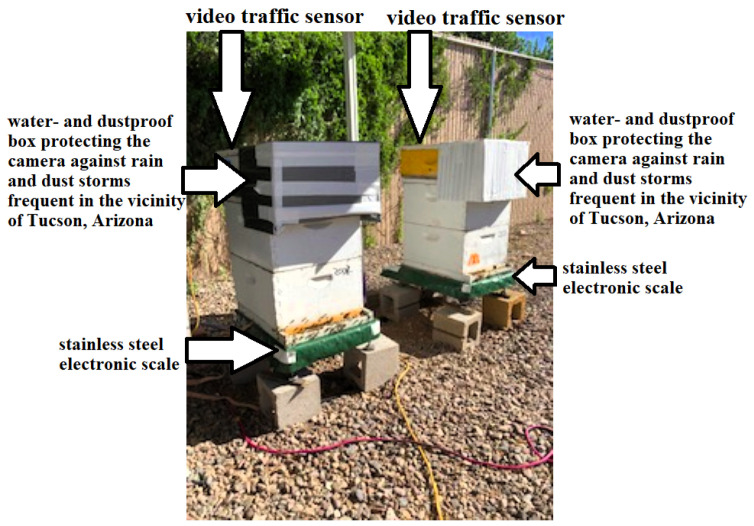
**Sensor Placement.** Weight and video sensors are external; temperature sensor–internal.

**Figure A4:**
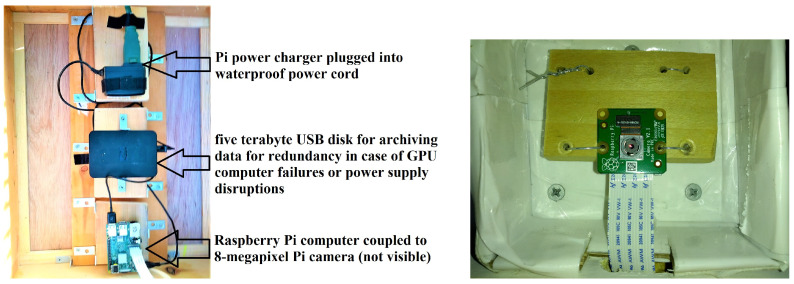
**Traffic sensor hardware. LEFT**: The hardware is placed into a separate wooden box on top of the hive and separated from the hive by an inner hive cover. **RiGHT**: A Raspberry Pi v2 camera (8 megapixel, 1080 × 1920 pix resolution) is connected to the Raspberry Pi computer; the camera looks down on the landing pad of the hive from the top of the second super in Figure A2.

**Figure A5:**
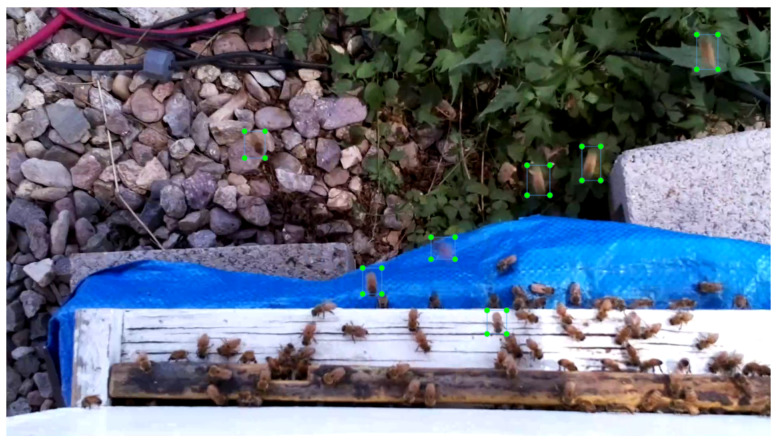
**Bee motion counts with OmniBeeM.** A frame from a video with detected flying bee objects (cf. Kulyukin and Kulyukin, 2023 [40] for the details of the bee traffic analysis algorithm).

## Data Availability

The file supp_mats.pdf contains the link to our FAIR dataset. The file ST.pdf included in the submission contains the supplementary tables discussed in Section 4.

## Supplementary Materials

The following supporting information can be downloaded at: https://www.mdpi.com/article/10.3390/s24196433/s1.

## Abbreviations

DUTS	discrete univariate time series
AI, DL, ML	artificial intelligence, deep learning, machine learning
ANN	ariificial neural network
CNN	convolutional neural network
LSTM	long short-term memory
ARIMA	Autoregressive Integrated Moving Average
FAIR	Findable, Accessible, Interoperable, Reusable
GPU, USB	graphical processing unit, universal serial bus
MP4, RGB,	MPEG-4, red green blue
YOLO	You Only Look Once
MSE	Mean Squared Error
RMSE	Root Mean Squared Error
CSV	Comma Separated Values
KNN	K Nearest Neighbors
USDA	U.S. Department of Agriculture
ARS	Agricultural Research Service
