# Supplementary material for: Discrete Time Series Forecasting of Hive Weight, In-Hive Temperature, and Hive Entrance Traffic in Non-Invasive Monitoring of Managed Honey Bee Colonies: Part I
Source: Sensors (Basel). 2024 Oct 4;24(19):6433. doi: 10.3390/s24196433 (PMC11479372; doi:10.3390/s24196433)
Supplement: Supplementary file 1 [file sensors-24-06433-s001.zip › ST.pdf]

Article

Discrete Time Series Forecasting of Hive Weight, In-Hive Temperature, and Hive Entrance Traffic in Non-Invasive Monitoring of Managed Honey Bee Colonies: Part I: Supplementary Tables

Vladimir A. Kulyukin[1], Daniel Coster[2], Aleksey V. Kulyukin[3], William Meikle[4], Milagra Weiss[4]

[1] Department of Computer Science, Utah State University, Logan, UT 84322, USA; vladimir.kulyukin@usu.edu (V.K.);
[2] Department of Mathematics and Statistics, Utah State University, Logan, UT 84322, USA; dan.coster@usu.edu (D.C.)
[3] Department of Data Analytics and Information Systems, Huntsman School of Business, Utah State University, Logan, UT 84322, USA; aleksey.kulyukin.2021@gmail.com (A.K.)
[4] Carl Hayden Bee Research Center, USDA-ARS, Tucson, AZ 85719, USA; william.meikle@usda.gov (W.M.); milagra.weiss@usda.gov (W.M.)
* Correspondence: vladimir.kulyukin@usu.edu

Abstract: This document contains additional tables for the Results Section of our article. In the main text of this article, the document is called **ST.pdf**. Each table in this document is referred to as **ST X**, e.g., **ST 7** in the main text of the article.

The PDF may have to be enlarged to see the plots.

Table 1. ST 1: IN=6 minimum MSE weight forecaster model Counts. Counts of times when an IN=6 weight forecaster model trained on the train data (70%) of a hive had minimum MSE on the test data (30%) of the same hive. The counts are reported for all 10 hives. Highest total counts are bolded.

IN	OUT	ANN	CNN	LSTM	ARIMA
6	1	1	0	0	9
6	2	0	0	0	10
6	3	0	0	0	10
6	4	1	0	0	9
6	5	1	0	0	9
6	6	0	0	2	8
TOT		3	0	2	**55**

Citation: Kulyukin, V.A.; Coster, D.; Kulyukin, A.V.; Meikle, W.; Weiss, M. Discrete Time Series Forecasting of Hive Weight, In-Hive Temperature, and Hive Entrance Traffic in Non-Invasive Monitoring of Managed Honey Bee Colonies: Part I: Supplementary Tables . *Sensors* **2024**, *1*, 0. https://doi.org/

Received:
Accepted:
Published:

Publisher's Note: MDPI stays neutral with regard to jurisdictional claims in published maps and institutional affiliations.

Copyright: © 2024 by the authors. Submitted to *Sensors* for possible open access publication under the terms and conditions of the Creative Commons Attri- bution (CC BY) license (https://creativecommons.org/licenses/by/4.0/).

Table 2. ST 2: IN=12 minimum MSE weight forecaster model counts. Counts of times when an IN=12 weight forecaster model trained on the train data (70%) of a hive had minimum MSE on the test data (30%) of the same hive. The counts are reported for all 10 hives. Highest total counts are bolded.

IN	OUT	ANN	CNN	LSTM	ARIMA
12	1	0	0	0	10
12	2	0	0	0	10
12	3	0	0	0	10
12	4	0	0	3	7
12	5	0	0	0	10
12	6	1	0	0	9
12	7	1	0	3	6
12	8	3	0	1	6
12	9	4	0	2	4
12	10	5	0	1	4
12	11	4	1	3	2
12	12	5	2	1	2
TOT		23	3	14	**80**

Table 3. ST 3: IN=6 minimum MSE in-hive temperature forecaster model counts. Counts of times when an IN=6 in-hive temperature forecaster model trained on the train data (70%) of a hive had minimum MSE on the test data (30%) of the same hive. The counts are reported for all 10 hives. Highest total counts are bolded.

IN	OUT	ANN	CNN	LSTM	ARIMA
6	1	0	0	4	6
6	2	0	0	7	3
6	3	0	0	9	1
6	4	0	1	8	1
6	5	0	0	9	1
6	6	1	0	9	0
TOT		1	1	**46**	12

Table 4. ST 4: IN=12 ninimum MSE in-hive temperature forecaster model counts. Counts of times when an IN=12 in-hive temperature forecaster model trained on the train data (70%) of a hive had minimum MSE on the test data (30%) of the same hive. The counts are reported for all 10 hives. Highest total counts are bolded.

IN	OUT	ANN	CNN	LSTM	ARIMA
12	1	0	0	2	8
12	2	0	0	3	7
12	3	0	0	5	5
12	4	0	0	9	1
12	5	0	0	8	2
12	6	0	0	8	2
12	7	2	0	8	0
12	8	3	0	7	0
12	9	2	0	8	0
12	10	5	1	4	0
12	11	4	1	5	0
12	12	6	0	4	0
TOT		22	2	**71**	25

Table 5. ST 5: IN=6 minimum MSE bee entrance traffic forecaster model counts. Counts of times when an IN=6 bee entrance traffic forecaster model trained on the train data (70%) of a hive had minimum MSE on the test data (30%) of the same hive. The counts are reported for all 10 hives. Highest total counts are bolded.

IN	OUT	ANN	CNN	LSTM	ARIMA
6	1	3	0	7	0
6	2	2	0	8	0
6	3	0	2	8	0
6	4	0	2	7	1
6	5	1	0	8	1
6	6	1	3	6	0
TOT		7	7	**44**	2

Table 6. ST 6: IN=12 minimum MSE bee entrance traffic forecaster model counts. Counts of times when an IN=12 bee entrance traffic forecaster model trained on the train data (70%) of a hive had minimum MSE on the test data (30%) of the same hive. The counts are reported for all 10 hives. Highest total counts are bolded.

IN	OUT	ANN	CNN	LSTM	ARIMA
12	1	1	1	6	2
12	2	10	0	0	0
12	3	9	1	0	0
12	4	9	0	1	0
12	5	9	1	0	0
12	6	10	0	0	0
12	7	7	3	0	0
12	8	9	1	0	0
12	9	9	1	0	0
12	10	9	1	0	0
12	11	9	1	0	0
12	12	10	0	0	0
TOT		**101**	10	7	2

Table 7. ST 7: Minimum MSE plots for best IN=6 and IN=12 weight forecasters. Minimum MSE plots of the best ANN, CNN, LSTM, and ARIMA IN=6 and IN=12 weight forecasters on the test data for hives 2059 and 2146.

**IN=6**	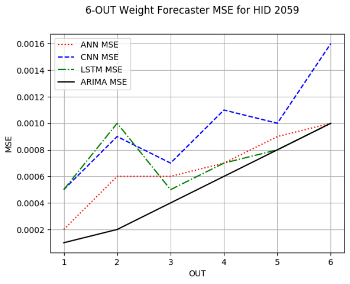	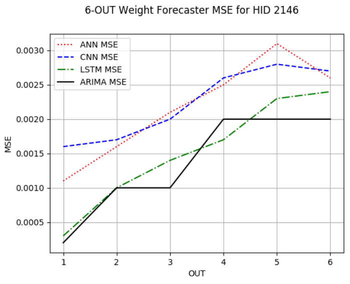
**IN=12**	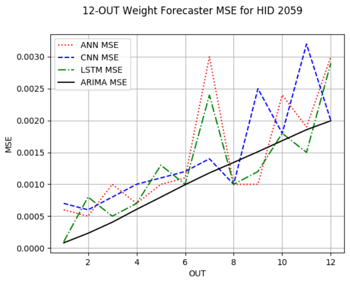	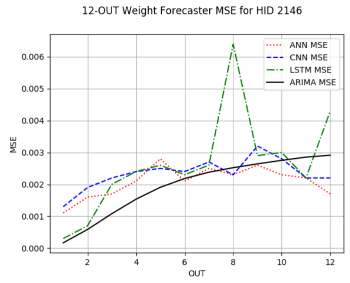

Table 8. ST 8: Minimum MSE plots for best IN=6 and IN=12 in-hive temperature forecasters. Minimum MSE plots of the best ANN, CNN, LSTM, and ARIMA IN=6 and IN=12 temperature forecasters on the test data for hives 2059 and 2146.

IN=6

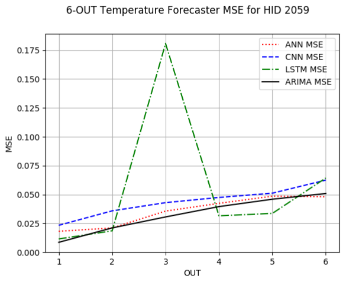

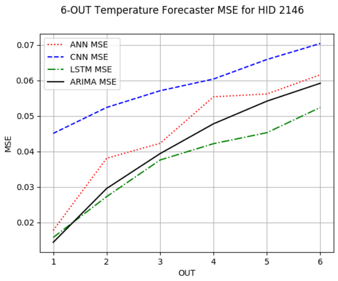

IN=12

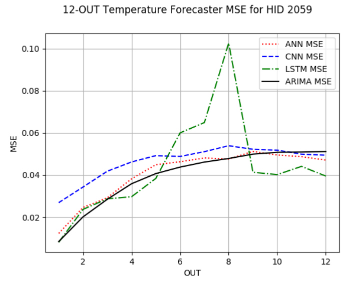

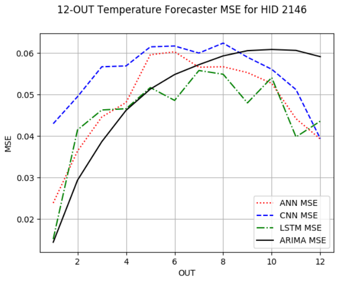

Table 9. ST 9: Minimum RMSE plots for IN=6 and IN=12 bee entrance traffic forecasters. Minimum RMSE plots of best IN=6 and IN=12 bee entrance traffic forecasters on the test data for hives 2059 and 2146. The omnidirectional bee counts are computed with OmniBeeM with YOLOv3.

IN=6

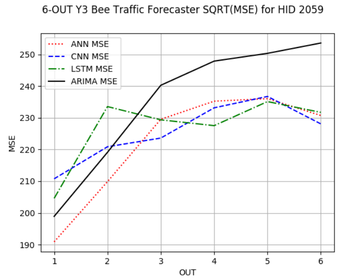

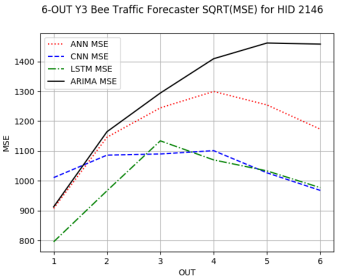

IN=12

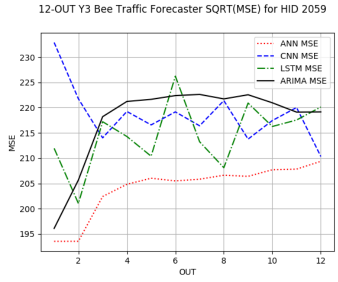

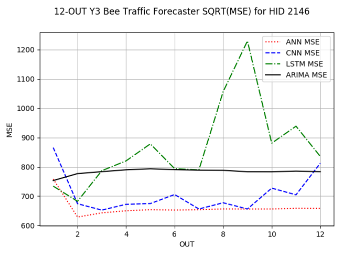

Table 10. ST 10: Observed vs. predicted MSE plots of best IN=6, OUT=1 and IN=6, OUT=6 weight forecasters for hive 2059.

	ANN	CNN	LSTM	ARIMA
**IN=6, OUT=1**	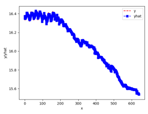	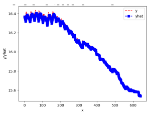	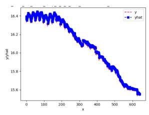	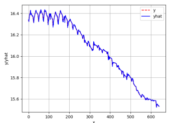
**IN=6, OUT=6**	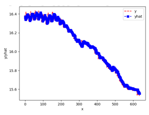	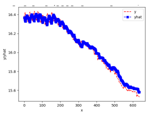	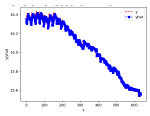	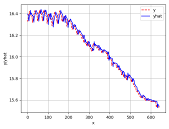

Table 11. ST 11: Observed vs. predicted MSE plots of best IN=6, OUT=1 and IN=6, OUT=6 weight forecasters for hive 2146.

	ANN	CNN	LSTM	ARIMA
**IN=6, OUT=1**	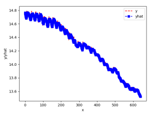	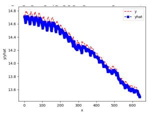	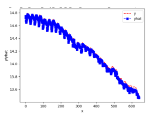	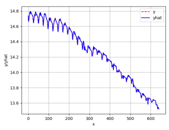
**IN=6, OUT=6**	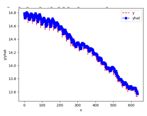	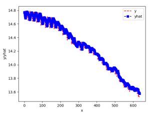	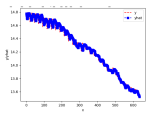	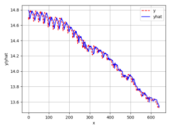

Table 12. ST 12: Observed vs. predicted MSE plots of best IN=12, OUT=1 and IN=12, OUT=12 weight forecasters for hive 2059.

	ANN	CNN	LSTM	ARIMA
**IN=12, OUT=1**	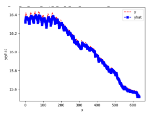	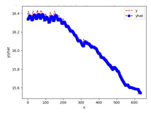	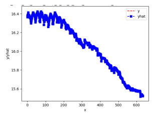	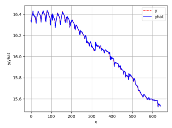
**IN=12, OUT=12**	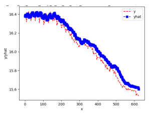	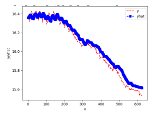	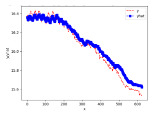	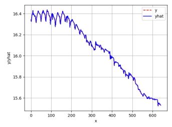

Table 13. ST 13: Observed vs. predicted MSE plots of best IN=12, OUT=1 and IN=12, OUT=12 weight forecasters for hive 2146.

	ANN	CNN	LSTM	ARIMA
**IN=12, OUT=1**	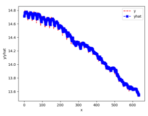	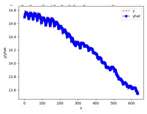	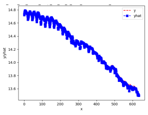	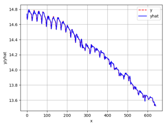
**IN=12, OUT=12**	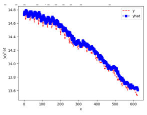	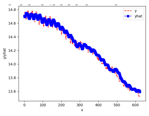	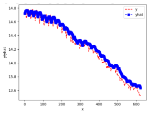	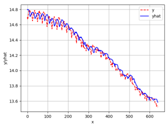

Table 14. ST 14: Observed vs. predicted MSE plots of best IN=6, OUT=1 and IN=6, OUT=6 in-hive temperature forecasters for hive 2059.

	ANN	CNN	LSTM	ARIMA
**IN=6, OUT=1**	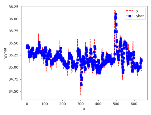	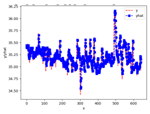	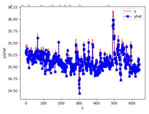	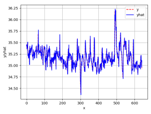
**IN=6, OUT=6**	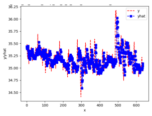	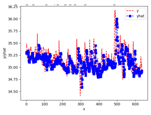	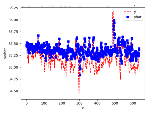	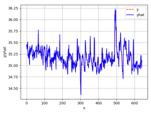

Table 15. ST 15: Observed vs. predicted MSE plots of best IN=6, OUT=1 and IN=6, OUT=6 in-hive temperature for hive 2146.

	ANN	CNN	LSTM	ARIMA
**IN=6, OUT=1**	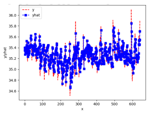	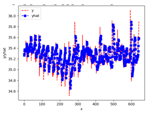	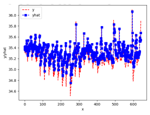	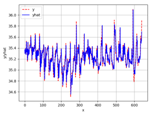
**IN=6, OUT=6**	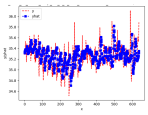	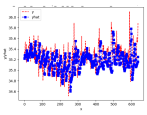	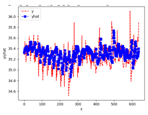	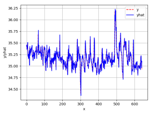

Table 16. ST 16: Observed vs. predicted MSE plots of best IN=12, OUT=1 and IN=12, OUT=12 in-hive temperature for hive 2059.

	ANN	CNN	LSTM	ARIMA
**IN=12, OUT=1**	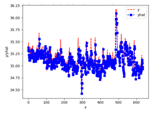	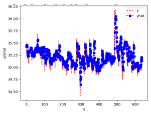	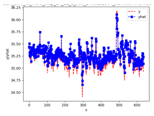	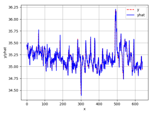
**IN=12, OUT=12**	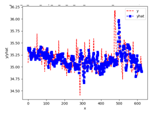	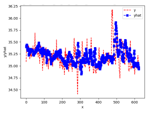	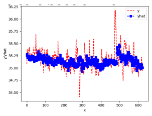	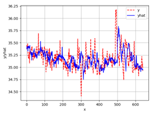

Table 17. ST 17: Observed vs. predicted MSE plots of best IN=12, OUT=1 and IN=12, OUT=12 in-hive temperature for hive 2146.

	ANN	CNN	LSTM	ARIMA
**IN=12, OUT=1**	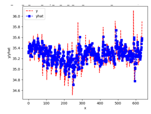	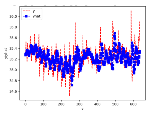	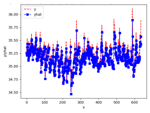	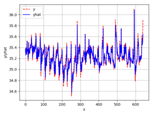
**IN=12, OUT=12**	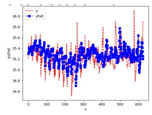	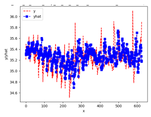	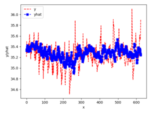	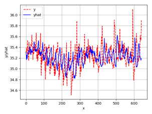

Table 18. ST 18: Observed vs. predicted MSE plots of best IN=6, OUT=1 and IN=6, OUT=6 bee entrance traffic forecasters for hive 2059.

	ANN	CNN	LSTM	ARIMA
**IN=6, OUT=1**	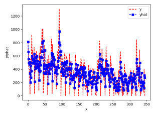	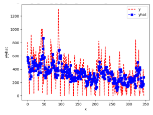	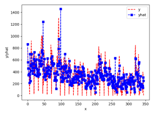	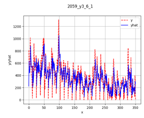
**IN=6, OUT=6**	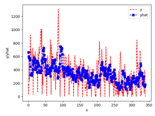	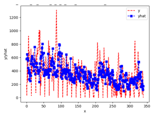	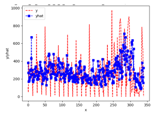	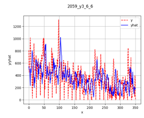

Table 19. ST 19: Observed vs. predicted MSE plots of best IN=6, OUT=1 and IN=6, OUT=6 bee entrance traffic for hive 2146.

	ANN	CNN	LSTM	ARIMA
**IN=6, OUT=1**	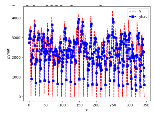	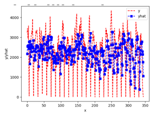	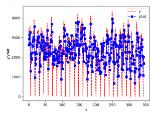	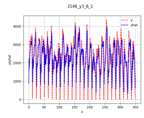
**IN=6, OUT=6**	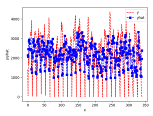	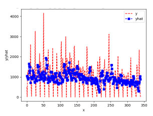	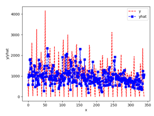	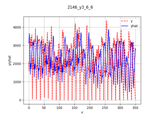

Table 20. ST 20: Observed vs. predicted MSE plots of best IN=12, OUT=1 and IN=12, OUT=12 bee entrance traffic for hive 2059.

	ANN	CNN	LSTM	ARIMA
**IN=12, OUT=1**	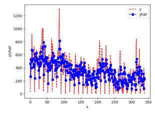	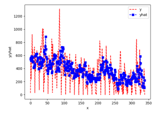	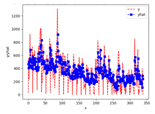	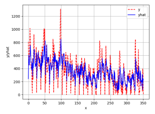
**IN=12, OUT=12**	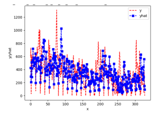	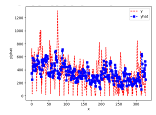	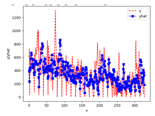	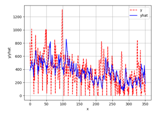

Table 21. ST 21: Observed vs. predicted MSE plots of best IN=12, OUT=1 and IN=12, OUT=12 bee entrance traffic for hive 2146.

**IN=12, OUT=1**	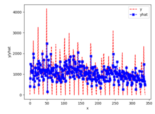	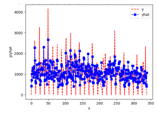	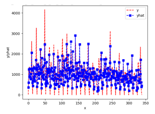	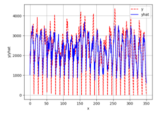
**IN=12, OUT=12**	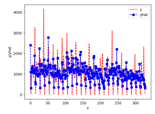	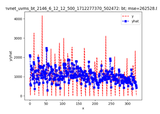	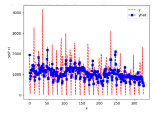	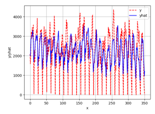
