# Supplementary material for: Discrete Time Series Forecasting of Hive Weight, In-Hive Temperature, and Hive Entrance Traffic in Non-Invasive Monitoring of Managed Honey Bee Colonies: Part I
Source: Sensors (Basel). 2024 Oct 4;24(19):6433. doi: 10.3390/s24196433 (PMC11479372; doi:10.3390/s24196433)
Supplement: Supplementary file 1 [file sensors-24-06433-s001.zip › supp_mats.pdf]

Article

Discrete Time Series Forecasting of Hive Weight, In-Hive Temperature, and Hive Entrance Traffic in Non-Invasive Monitoring of Managed Honey Bee Colonies: Part I: Supplementary Materials

Vladimir A. Kulyukin[1], Daniel Coster [2], Aleksey V. Kulyukin[3], William Meikle[4], Milagra Weiss[4]

[1] Department of Computer Science, Utah State University, Logan, UT 84322, USA
[2] Department of Mathematics and Statistics, Utah State University, Logan, UT 84322, USA
[3] Department of Data Analytics and Information Systems, Huntsman School of Business, Utah State University, Logan, UT 84322, USA
[4] Carl Hayden Bee Research Center, USDA-ARS, Tucson, AZ 85719, USA
* Correspondence: vladimir.kulyukin@usu.edu

Abstract: This document contains a brief description of the supplementary materials to our article.

The supplementary materials to this article can be found at https://usu.box.com/s/9orz49spipz4nx89j2b2lqrzd0j7p3co.

The archive contains the README with a description of its contents. The archive contains 4 folders: `DATA`, `MSE_PLOTS`, `SOURCE_CODE`, `TRAINED_MODELS`.

The `DATA` folder contains the folder `WT_TH_BT_HOURLY_MEANS` which contains CSV files on which we trained our ANN, CNN, LSTM, and ARIMA models with a 70/30 train/test split. The files WT_TH_HOURLY_MEANS.csv contains the time stamped hourly weight and temperature measurements for each of the 10 hives.

The files contain the raw and mean entrance bee traffic measurements for each of the 10 hives. The files contain the measurements of our OmniBeeM algorithm with YOLO 3, YOLO 4 Tiny, and YOLO 7 Tiny.

`h2059allyolo.csv`	`h2129yolomeans.csv`	`h2142yolomeans.csv`
`h2059yolomeans.csv`	`h2130allyolo.csv`	`h2146allyolo.csv`
`h2130yolomeans.csv`	`h2146yolomeans.csv`	`h2120allyolo.csv`
`h2137allyolo.csv`	`h2158allyolo.csv`	`h2120yolomeans.csv`
`h2137yolomeans.csv`	`h2158yolomeans.csv`	`h2123allyolo.csv`
`h2141allyolo.csv`	`h2123yolomeans.csv`	`h2141yolomeans.csv`
`h2129allyolo.csv`	`h2142allyolo.csv`	

The folder BT_CSV data duplicates the above CSV files for each hive. Our univariate time series forecasting models of colony entrance traffic were trained on the means CSV files for each hive with a 70/30 train/test split.

The MSE Plots contains the MSE plots for ANN, CNN, LSTM, and ARIMA models and/or CSV files from which one can generate these plots.

SOURCE_CODE contains the Python source for the ANN, CNN, LSTM forecasters and the SAS source of our ARIMA forecasters.
